# Lamin B1 Polymorphism Influences Morphology of the Nuclear Envelope, Cell Cycle Progression, and Risk of Neural Tube Defects in Mice

**DOI:** 10.1371/journal.pgen.1003059

**Published:** 2012-11-15

**Authors:** Sandra C. P. De Castro, Ashraf Malhas, Kit-Yi Leung, Peter Gustavsson, David J. Vaux, Andrew J. Copp, Nicholas D. E. Greene

**Affiliations:** 1 Neural Development Unit, UCL Institute of Child Health, University College London, London, United Kingdom; 2 Sir William Dunn School of Pathology, University of Oxford, Oxford, United Kingdom; 3 Department of Molecular Medicine and Surgery, Karolinska Institute, Stockholm, Sweden; University of California Irvine, United States of America

## Abstract

Neural tube defects (NTDs), including spina bifida and anencephaly, are common birth defects whose complex multigenic causation has hampered efforts to delineate their molecular basis. The effect of putative modifier genes in determining NTD susceptibility may be investigated in mouse models, particularly those that display partial penetrance such as *curly tail*, a strain in which NTDs result from a hypomorphic allele of the *grainyhead-like-3* gene. Through proteomic analysis, we found that the *curly tail* genetic background harbours a polymorphic variant of lamin B1, lacking one of a series of nine glutamic acid residues. Lamins are intermediate filament proteins of the nuclear lamina with multiple functions that influence nuclear structure, cell cycle properties, and transcriptional regulation. Fluorescence loss in photobleaching showed that the variant lamin B1 exhibited reduced stability in the nuclear lamina. Genetic analysis demonstrated that the variant also affects neural tube closure: the frequency of spina bifida and anencephaly was reduced three-fold when wild-type lamin B1 was bred into the *curly tail* strain background. Cultured fibroblasts expressing variant lamin B1 show significantly increased nuclear dysmorphology and diminished proliferative capacity, as well as premature senescence, associated with reduced expression of cyclins and *Smc2*, and increased expression of *p16*. The cellular basis of spinal NTDs in *curly tail* embryos involves a proliferation defect localised to the hindgut epithelium, and S-phase progression was diminished in the hindgut of embryos expressing variant lamin B1. These observations indicate a mechanistic link between altered lamin B1 function, exacerbation of the *Grhl3*-mediated cell proliferation defect, and enhanced susceptibility to NTDs. We conclude that lamin B1 is a modifier gene of major effect for NTDs resulting from loss of *Grhl3* function, a role that is likely mediated via the key function of lamin B1 in maintaining integrity of the nuclear envelope and ensuring normal cell cycle progression.

## Author Summary

Failure of early development of the central nervous system leads to severe malformations termed neural tube defects (NTDs), including spina bifida and anencephaly. Inherited genetic risk factors play a major role in determining susceptibility to NTDs, but causative genes have proven difficult to identify. In this study we investigated genetic factors that could alter the risk of NTDs in an established mouse model, *curly tail*, in which defects result from partial loss of function of the *grainyhead-like-3* (*Grhl3*) gene. We identified a variant of lamin B1, a key protein component of the envelope that surrounds the cell nucleus. The protein alteration reduces the structural integrity of the nuclear envelope, causes the nuclei to have altered shape, and reduces the rate of cell division. *Curly tail* embryos that carry the “abnormal” lamin B1 variant develop NTDs at three times the rate of those that carry the normal version. We conclude that lamin B1 function influences risk of NTDs due to effects on cell proliferation.

## Introduction

Modifier genes have been ascribed significant influence in determining susceptibility to disease in complex traits, as well as partial penetrance and variable expressivity of monogenic conditions [1]. Moreover, modifier genes are considered largely responsible for the phenotypic variation observed when mutations are bred onto different genetic backgrounds in mice. However, identification of modifier genes and determination of their functional effects presents a considerable challenge. Understanding the genetic basis of neural tube defects (NTDs), such as spina bifida and anencephaly, typifies these difficulties.

NTDs are common, severe congenital malformations resulting from failure of closure of the neural tube during embryonic development [2]. In humans, they are among the commonest birth defects, affecting around 1 per 1000 pregnancies worldwide. However, the causes are not well understood owing to their multigenic inheritance and the potential influence of environmental factors, either predisposing or ameliorating [3], [4]. The potential complexity of NTD genetics is illustrated by the fact that more than 200 different genes have been implicated as potential contributors to the overall burden of NTDs, with neural tube closure phenotypes in mouse strains carrying naturally occurring or targeted mutations [5]–[7]. Additionally, in many of these models penetrance is influenced by genetic background, indicating the presence of modifier genes.

The *curly tail* (*ct*) mouse mutant is among the most extensively characterised models of NTDs [8]. Approximately 5–10% of homozygous *ct/ct* embryos develop cranial NTDs (exencephaly), while 15–20% exhibit spinal NTDs (spina bifida), due to failure of closure of neural folds in the prospective brain and low spinal region, respectively. The major *ct* gene corresponds to a hypomorphic allele of the transcription factor *grainyhead-like-3* (*Grhl3*), null mutants of which display spina bifida with 100% penetrance [9]–[11]. Expression of *Grhl3* is diminished in the hindgut of *ct* mutant embryos, due to an upstream regulatory mutation, resulting in a diminished cellular proliferation rate in the hindgut endoderm [12], [13]. The consequent dorso-ventral growth imbalance leads to excessive ventral curvature of the caudal region of the embryo and, hence, mechanical suppression of neural tube closure at the posterior neuropore [14]. The incidence of *curly tail* NTDs can be influenced by multiple environmental and genetic factors [8], [15]–[18]. In addition, NTD frequency is also markedly affected by backcross to different strains, indicating the presence of modifier loci in the *curly tail* genetic background [19]. Thus, it is apparent that the genetic component of predisposition to NTDs is multifactorial in *ct*, as in humans.

In the current study, we identified *lamin B1* as a modifier gene for NTDs in *curly tail* mice. Lamins are intermediate filament proteins of which the A-type, lamins A and C, are encoded by *LMNA* while, among the B-type, lamin B1 is encoded by *LMNB1* and lamins B2 and B3, are encoded by *LMNB2*. The nuclear lamina is a protein complex underlying the inner nuclear membrane and composed of a meshwork of lamin polymers and lamin-binding proteins [20]–[22]. In addition to a key structural role in assembly and maintenance of the nuclear envelope, it has become clear that lamins have multiple functions in a diverse range of cellular properties. Thus, lamins influence nuclear shape and size as well as anchoring of protein structures, including nuclear pore complexes, in the nuclear envelope [23], [24]. Additionally, lamins function in DNA synthesis and transcriptional regulation both through interaction with chromatin, to mediate sub-nuclear chromosomal positioning, and by direct interactions with transcription factors [25]–[29].

Highlighting the importance of lamin function, a number of clinically distinct diseases, termed laminopathies, have been found to result from mutation of *LMNA* [20], [30]. These include muscular dystrophy disorders (e.g. Emery-Dreyfus muscular dystrophy), lipodystrophies, progeria syndromes (e.g. Hutchinson-Gilford progeria syndrome and Atypical Werner syndrome) and peripheral neuropathy (Charcot-Marie-Tooth disease type 2B1). In contrast to *LMNA*, coding mutations in *LMNB1* have not so far been associated with human disease, although genomic duplication of *LMNB1* is thought to cause a progressive demyelinating disorder, adult-onset autosomal dominant leukodystrophy [31], [32]. Mice homozygous for a loss of function allele of *Lmnb1* die at birth with reduced growth, impaired lung development and cortical abnormalities in the brain [33], [34], while *Lmnb2* knockouts exhibit neuronal migration defects in the cerebral cortex and cerebellum [35]. *Lmnb1/lmnb2* double knockouts exhibit a reduced thickness of the brain cortex, with altered cell cycle exit of neuronal progenitors and neuronal migration defects [29], [34]. Forebrain-specific deletion of *lmnb1* or *lmnb2*, allowed study of brain phenotypes at post-natal stages and showed that both genes are individually required for normal development of the cortex [34].

In the current study we identified a polymorphic variant form of lamin B1, present on the genetic background of the *curly tail* strain. The reduction in length of a series of glutamic acid residues, from nine to eight, was found to cause significant reduction in the stability of the lamin B1 interaction within the nuclear lamina. Genetic analysis, involving generation of *curly tail* sub-strains carrying combinations of the lamin B1 variant and *Grhl3^ct^* mutation demonstrate a dramatic effect of lamin B1 on frequency of NTDs. In parallel, lamin B1 has a profound effect on nuclear morphology and proliferative capacity. Overall, our findings show that *Lmnb1* can act as a modifier gene affecting risk of NTDs, an effect that appears to be mediated through impaired cell cycle regulation which summates with the effect of *Grhl3* mutation.

## Results

**Figure 1:**
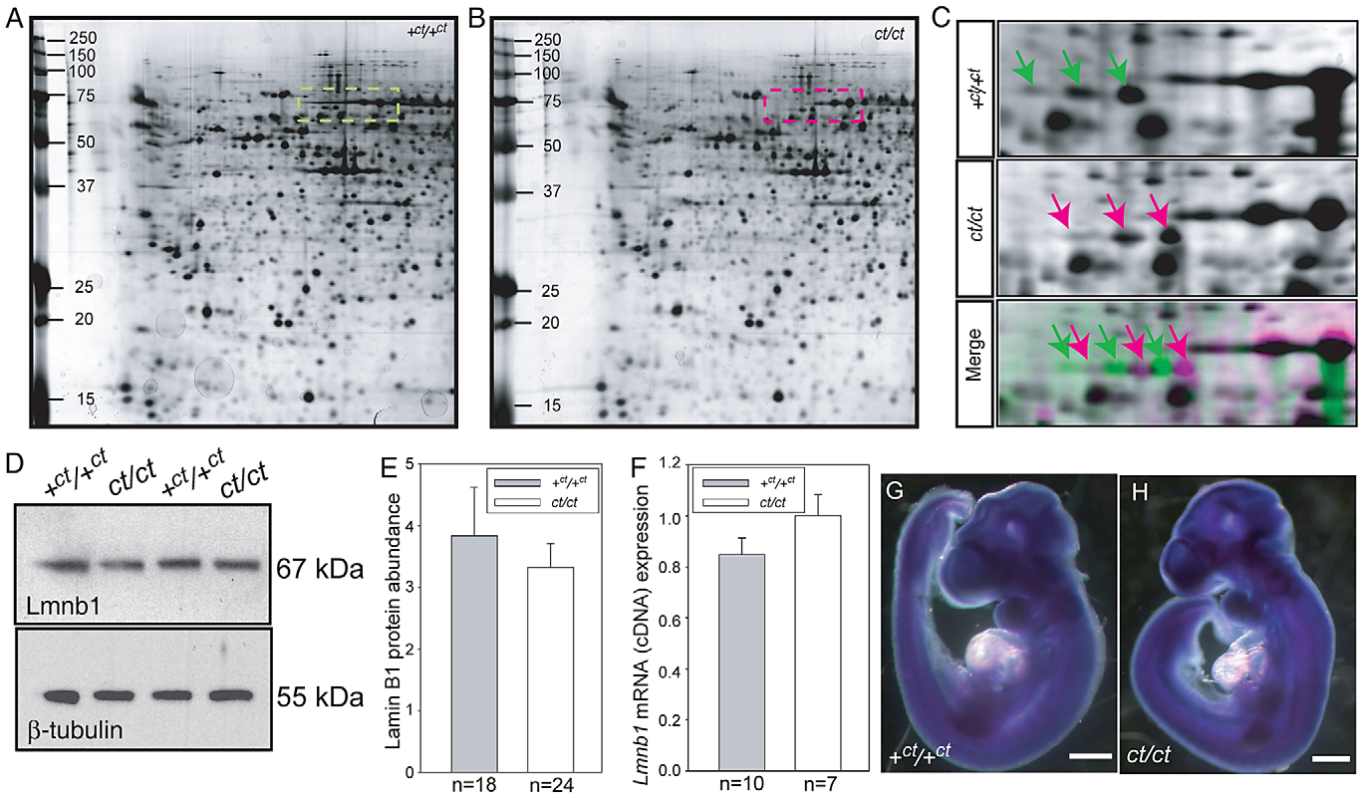
Lamin B1 shows differential protein migration by two-dimensional gel electrophoresis in *curly tail* and wild-type embryo samples. Protein profiles of the caudal region of stage-matched wild-type (A) and *curly tail* (B) embryos analysed by 2-DE (representative gels shown encompass pH 3.0–5.6 on the x-axis, basic pH to the right). A series of silver-stained protein spots exhibits a different migration pattern (C: enlarged area of gel corresponds to dashed box in A and B). On 2-DE of *ct/ct* samples, spots (pink arrows and pink spots on merged image) migrate to a more basic position than the corresponding spots on +*^ct^*/+*^ct^* gels (green arrows and green spots on merged image). Spots whose migration does not differ between samples appear black on the merged image. Western blot (D–E) and qRT-PCR (F) of protein and mRNA samples from the caudal region of *+^ct^/+^ct^* and *ct/ct* embryos at E10.5 show no significant difference between strains in relative abundance of either lamin B1 protein (normalised to beta-tubulin; arbitrary units) or mRNA (normalised to *Gapdh* with one wild-type sample chosen as calibrator; value set to 1.0). Number of samples, n, is shown on graphs. (G, H) The distribution of *Lmnb1* mRNA at E10.5, as determined by whole mount *in situ* hybridisation is comparable in wild-type and *ct/ct* embryos (scale bar represents 1 mm).

In a proteomic analysis of the *curly tail* mutant, two-dimensional protein gels were generated from samples at embryonic day (E) 10.5: the stage of spinal neural tube closure. Comparison of stage-matched embryos revealed differential migration of a series of three spots, which migrated to a more basic position in gels derived from *ct/ct* samples than the equivalent spots in congenic wildtype (*+^ct^/+^ct^*) control gels (Figure 1A–1C). This migration change was apparent by the complete absence of the three spots that were detected in the *+^ct^/+^ct^* gels from the *ct/ct* gels and vice versa. This difference was detected both in analysis of whole embryos and in isolated caudal regions that encompassed the posterior neuropore (PNP), the region of active neural tube closure. In both strains, these spots were identified by liquid chromatography tandem mass spectrometry as lamin B1 (Table S1). Variation in abundance of some other spots between genotypes was observed, however, no spots other than those corresponding to lamin B1 showed a difference in migration. Neither the abundance of *Lmnb1* mRNA nor total lamin B1 protein abundance were found to differ between *ct/ct* and +*^ct^/+^ct^* embryos, by real time qRT-PCR or western blot respectively (Figure 1D–1F). Moreover, the sites of *Lmnb1* expression at neurulation stages were also comparable between genotypes as determined by whole mount *in situ* hybridisation (Figure 1G–1H). Expression was apparent throughout most of the embryo with the exception of surface ectoderm and the heart (Figure S1), where staining intensity was much lower than in other tissues.

**Figure 2:**
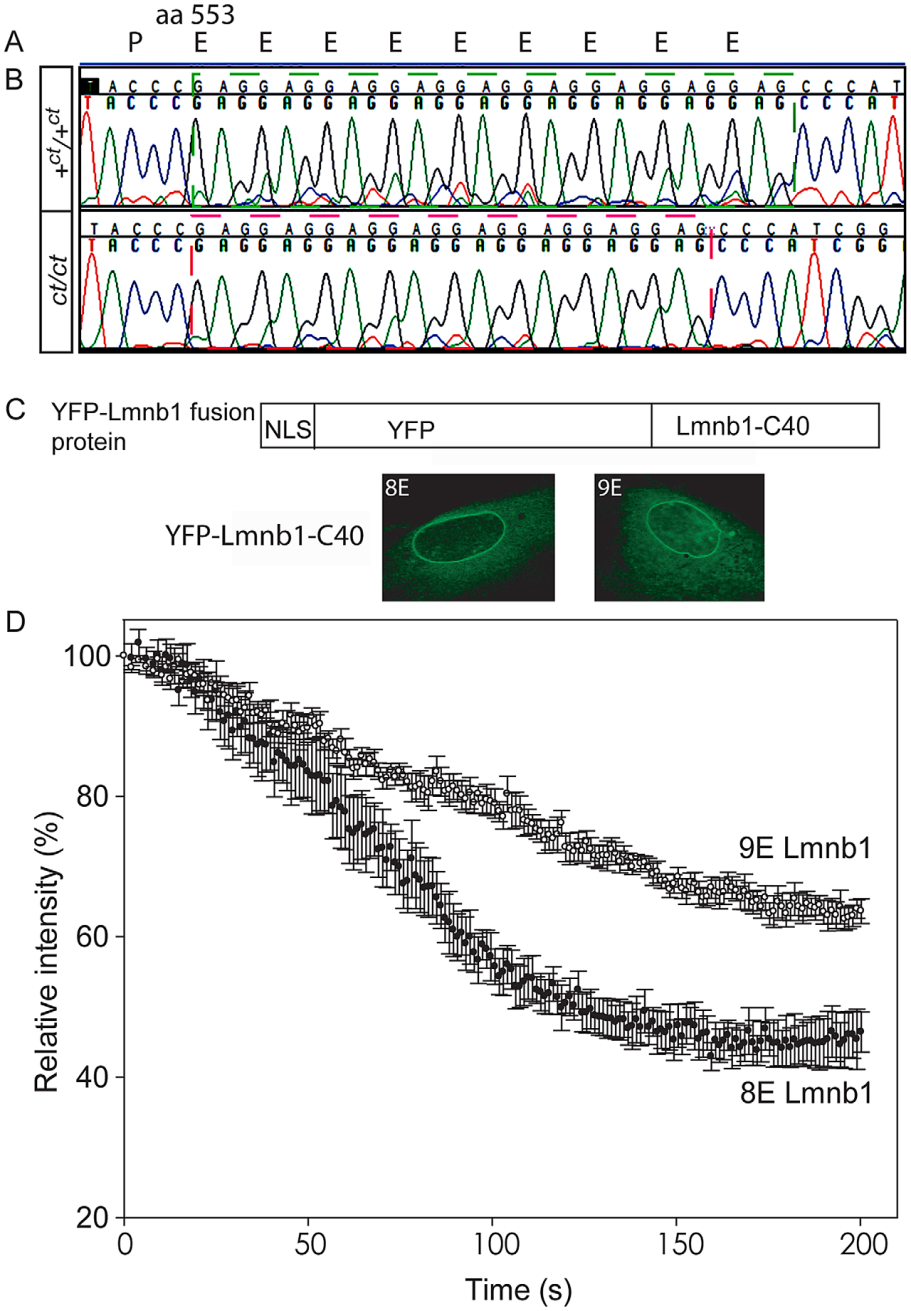
Lamin B1 shows variation in length of glutamic acid repeat, which significantly affects mobility within the nuclear envelope. (A) Lamin B1 protein sequence contains a series of Glu (E) residues beginning at amino acid 553, encoded at the genomic level by a GAG repeat. (B) The wild-type (+*^ct^*) sequence encodes nine E residues, whereas the *curly tail* sequence encodes only eight E residues (one fewer GAG). (C–D) Fluorescence loss in photobleaching (FLIP) was performed to examine the mobility of NLS-YFP-Lamin B1 tail domain fusion proteins, containing eight or nine Glu residues. (C) Fusion proteins are shown diagrammatically and localised to the nuclear envelope as expected. (D) The relative intensity of fluorescence in an unbleached region of the nuclear envelope compared with pre-bleaching (time 0) was determined. Mean intensity ± SEM is shown for five nuclei with each construct. There is a more rapid decline in intensity in cells expressing the 8E variant (red circles) compared with the 9E variant (open circles), and differences persist throughout the experiment. Statistical analysis shows a significant difference between the 8E and 9E variants at both the 100 s and 200 s time points (*p*<0.001, t-test).

Altered migration of lamin B1 during the isoelectric focussing step of 2-DE results from a charge difference between the protein in *ct/ct* and *+^ct^/+^ct^* samples. Such a difference could potentially result from an alteration in primary sequence and the *Lmnb1* coding region was therefore sequenced in *ct/ct* and *+^ct^/+^ct^* genomic DNA and cDNA. A synonymous polymorphism, C612T (annotated as SNP 18: 56868078), was found in exon 1 of the *ct/ct* sequence. In addition, a three base-pair GAG deletion (annotated as Deletion 18: 56909394) was noted in exon 10. This deletion corresponds to one of a sequence of GAG nucleotides at position 1657–1683 of the coding sequence, encoding a stretch of nine glutamic acid (Glu) residues in the tail domain of the wild-type protein (Figure 2A, 2B). Thus, the *curly tail Lmnb1* gene encodes eight Glu residues at amino acids 553–560 (here denoted *Lmnb1^8E^* to indicate number of glutamic acids), as opposed to nine Glu (residues 553–561) encoded by the +*^ct^/+^ct^* sequence (denoted *Lmnb1^9E^*). Since Glu carries a negative charge, it appeared likely that the difference in number of Glu residues is responsible for the migration difference of lamin B1 spots on 2D gels generated from *ct/ct* and +*^ct^/+^ct^* samples.

The Glu repeat in the lamin B1 tail domain is predicted to form an alpha-helix (PSIPRED secondary structure prediction [36]). Loss of a residue would impose a hundred degree rotation on the C-terminal region of the protein. The helix is likely to be capable of interacting with the inner nuclear phospholipid membrane [37]. Given that this region contains another strong membrane interactor, the C-terminal farnesylcysteine, we hypothesised that the interaction of lamin B1 with the nuclear membrane could be affected by variation in the number of Glu residues. We therefore used fluorescence loss in photobleaching (FLIP) to investigate possible functional effects on the stability of the lamin B1 tail domain within the nuclear envelope. Full length laminB1-YFP fusion proteins appeared to be stably integrated into the nuclear lamina without apparent difference between variants. We also tested truncated forms of the protein as these have previously been found to provide greater sensitivity to altered properties in this assay [26]. Fusion proteins comprising a nuclear localisation sequence, YFP and the forty C-terminal residues of lamin B1 were expressed in primary mouse embryonic fibroblasts (MEFs) and subjected to FLIP, as previously performed for human lamin B1 [26]. The decline in fluorescence intensity in the unbleached area of membrane was much more rapid in cells expressing Lmnb1^8E^ compared with Lmnb1^9E^ (Figure 2C). After 100 seconds, there was an approximately 43% decline in intensity in cells expressing Lmnb1^8E^ compared with only a 21% decline with Lmnb1^9E^ (*p*<0.001, t-test). This significant difference between variants persisted throughout the analysis, and is indicative of increased mobility, and hence decreased stability of interaction of Lmnb1^8E^ within the nuclear envelope.

Sequencing of exon 10 of lamin B1 in a series of mouse strains showed that the wild-type (Lmnb1^9E^) variant of lamin B1 is found in the majority of strains including C57BL/6, C3H/HeJ, SWR, DBA/2J, BALB/c, LPT/Le and CAST/EiJ. However, the Lmnb1^8E^ variant occurs in CBA/Ca, a sub-strain of which (CBA/Gr) contributed to the genetic background of the *curly tail* strain [38]. The variant was also present in the 101 strain and hence in mice harbouring the *splotch* (*Sp^2H^*; *Pax3*) mutation, which arose in a mutagenesis experiment on a mixed CBA/101 genetic background [39]. The 18: 56868078 SNP and Deletion 18: 56909394 were found to be in linkage disequilibrium. Thus, the Lmnb1^8E^ variant in *ct/ct* is characteristic of this particular genetic background.

Embryos of the CBA/Ca strain do not exhibit developmental abnormalities under normal laboratory conditions, indicating that the Lmnb1^8E^ variant alone is insufficient to cause NTDs. Nevertheless, given the possible effect on stability of the lamina, we speculated that this variant could represent one of the modifier genes that are major determinants of penetrance of the *curly tail* defect. To test this idea, we inter-crossed *ct/ct* and +*^ct^/+^ct^* mice to generate sub-strains of mice carrying different combinations of the *Lmnb1* variant (i.e. *Lmnb1^8E^* and *Lmnb1^9E^*; abbreviated hereafter as *L^8E^* and *L^9E^*) and the *Grhl3* mutant allele (*Grhl3^ct^* or *Grhl3^+^*; abbreviated as *G^ct^* and *G^+^*). Each sub-strain was maintained in homozygous form for both *Lmnb1* and *Grhl3* alleles, that is: (i) *L^8E/8E^*; *G^ct/ct^* (denoted *ct^8E^*); (ii) *L^8E/8E^*; *G^+/+^* (denoted +*^ct;8E^*); (iii) *L^9E/9E^*; *G^ct/ct^* (denoted *ct^9E^*); (iv) *L^9E/9E^*; *G^+/+^* (denoted +*^ct;9E^*). In the +*^ct^* strain the genetic background is approximately 97% *curly tail* [9] and in each sub-strain it is predicted to be 99.5% *curly tail* (see Figure S2 for breeding scheme). Embryos were collected at E11.5–15.5 and analysed for the presence or absence of NTDs.

**Figure 3:**
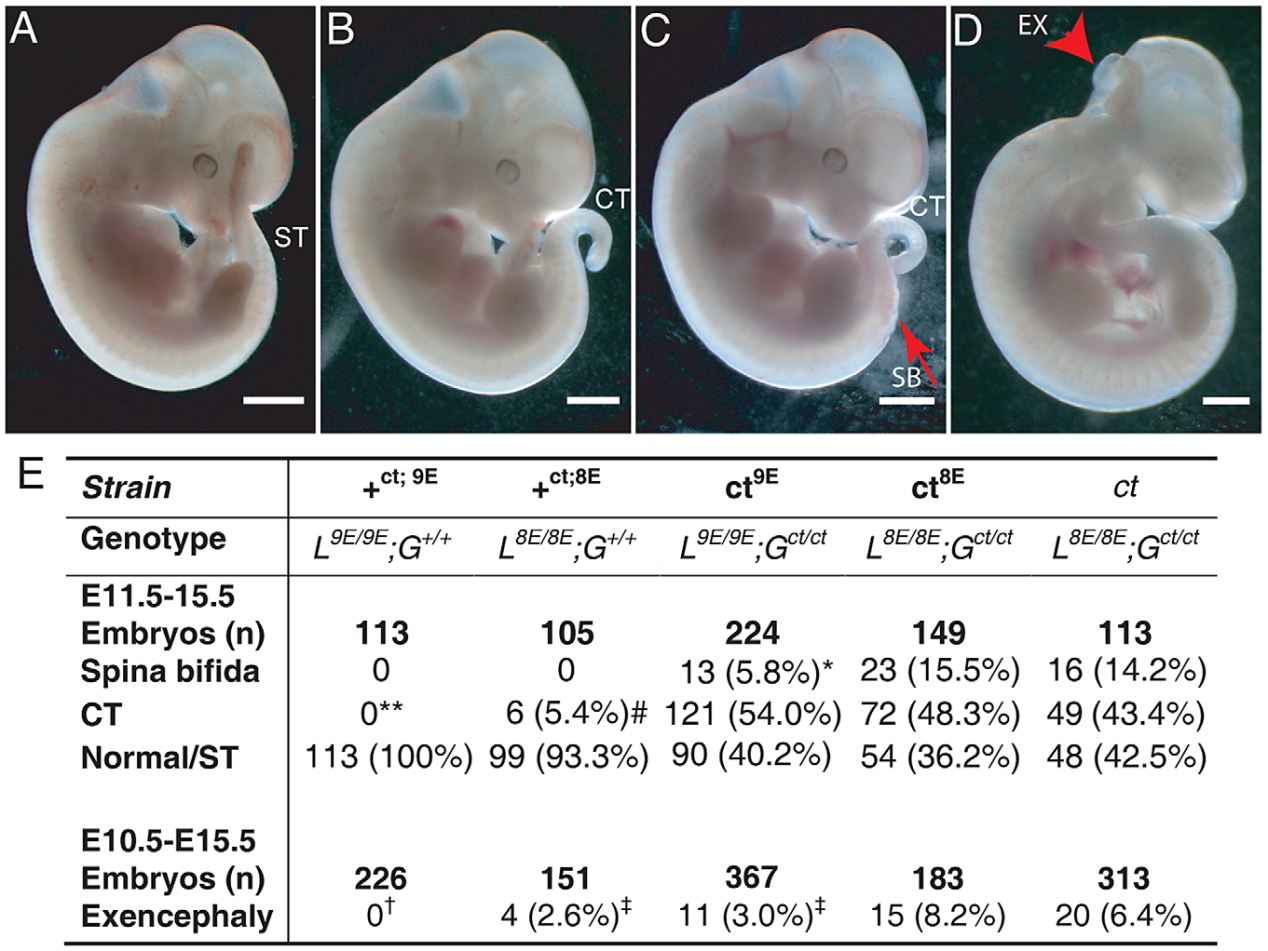
The frequency of NTDs resulting from mutation of *Grhl3* is affected by *Lmnb1* genotype. Embryos were scored as (A) apparently normal with a straight tail (ST), or with (B) a tail flexion defect (i.e. curly tail; CT), (C) spina bifida (SB) plus tail flexion defect, and/or (D) exencephaly (arrowhead indicates open hindbrain). Note that exencephaly can occur in association with any of the spinal phenotypes. Embryos shown are from the *ct^9E^* sub-strain. The frequency of NTD phenotypes is tabulated (E). The frequency of SB is significantly lower in the *ct^9E^* than in the *ct^8E^* and *ct* strains (* *p*<0.02, χ^2^ test). Spina bifida and tail flexion defects were never observed among *+^ct;9E^* embryos but tail flexion defects did occasionally occur among *+^ct;8E^* embryos (** *+^ct;9E^* versus *+^ct;8E^*; *p*<0.05; Z-test), although at significantly lower frequency than among *ct* mutant embryos (# *p*<0.001; χ^2^). There is significant variation in the frequency of exencephaly between the sub-strains (*p*<0.001; χ^2^) with a significantly lower exencephaly rate among *ct^9E^* than *ct^8E^* embryos (^‡^ significantly different from *ct^8E^*, *p*<0.01; Z-test). Exencephaly was not observed in the +*^ct;9E^* strain († indicates significant difference from *ct* and *ct^8E^* (*p*<0.001; Z-test) and *ct^9E^* (*p*<0.02) strains). Exencephaly was observed in the *+^ct;8E^* strain, albeit at a significantly lower frequency than in the *ct^8E^* strain (^‡^ *p*<0.05; Z-test).

Among embryos of the *ct^8E^* sub-strain, the range and frequency of phenotypes was closely similar to that observed in the *curly tail* (*ct*) strain which has the same genotype at the *Lmnb1* and *Grhl3* loci. Defects included spina bifida, tail flexion defects and exencephaly (Figure 3B–3D), while other embryos appeared normal (Figure 3A). Importantly, however, varying the *Lmnb1* genotype produced a striking difference in frequency of NTDs (Figure 3E). Thus, spina bifida occurred at significantly lower frequency in the *ct^9E^* sub-strain (5.8%) than in *curly tail* (14.2%) or in the *ct^8E^* sub-strain (15.8%). Therefore, homozygosity for the *Lmnb1^8E^* variant confers approximately three-fold higher risk of spina bifida in *G^ct/ct^* embryos, compared with homozygosity for the *Lmnb1^9E^* variant. Interestingly, although cranial NTDs occur at lower frequency than spina bifida in the *curly tail* strain, we also observed the rate of exencephaly to be significantly reduced among *ct^9E^* embryos (3.0%) compared with *curly tail* (6.4%) or *ct^8E^* embryos (8.2%). As expected, the frequency of exencephaly in the latter two strains did not differ significantly (Figure 3E).

Although the genetic background of each sub-strain was predicted to be approximately 99.5% *curly tail* we could not exclude a possible effect of the region of DNA that is tightly linked and inherited with *lmnb1*. We therefore examined the possibility that a neighbouring gene to *lmnb1* could vary in expression between the *ct^9E^* and *ct^8E^* sub-strains. Using a list of genes that are located within a 41 Mb interval of chromosome 18 centred on *lmnb1*, we interrogated microarray data generated from RNA of the caudal region of stage-matched *ct/ct* and +*^ct^*/+*^ct^* embryos (E10.5; 28–29 somite stage). Among 11 differentially expressed genes (p<0.05; fold-change 1.5-fold or greater), 4 showed a similar trend of differential expression on qRT-PCR analysis of independent *ct/ct* and +*^ct^*/+*^ct^* samples. However, none of these genes varied in expression when analysed by qRT-PCR in stage-matched *ct^9E^* and *ct^8E^* samples (Table S2), suggesting that the phenotypic difference between the sub-strains does not result from differential expression of genes located in proximity to *lmnb1*. Instead, variation in expression between *ct/ct* and +*^ct^*/+*^ct^* samples seem likely to be due to downstream effects of the *Grhl3^ct^* mutation in *ct/ct* embryos.

Embryos of the wild-type congenic *curly tail* strain (+*^ct;9E^*, genotype: *L^9E/9E^; G^+/+^*) do not develop exencephaly, spina bifida or tail flexion defects (Figure 3E) [9]. However, when the *Lmnb1^8E^* variant was bred onto the *Grhl3* wild-type genetic background, to produce mice of *L^8E/8E^; G^+/+^* genotype (+*^ct;8E^*), we observed a low frequency of tail flexion defects, indicative of delayed PNP closure (Figure 3E). Exencephaly was also occasionally observed (Figure 3E). These data demonstrate that the presence of the *Lmnb1^8E^* variant can predispose to defects of cranial and spinal neural tube closure, even in the absence of the *Grhl3* mutation.

**Figure 4:**
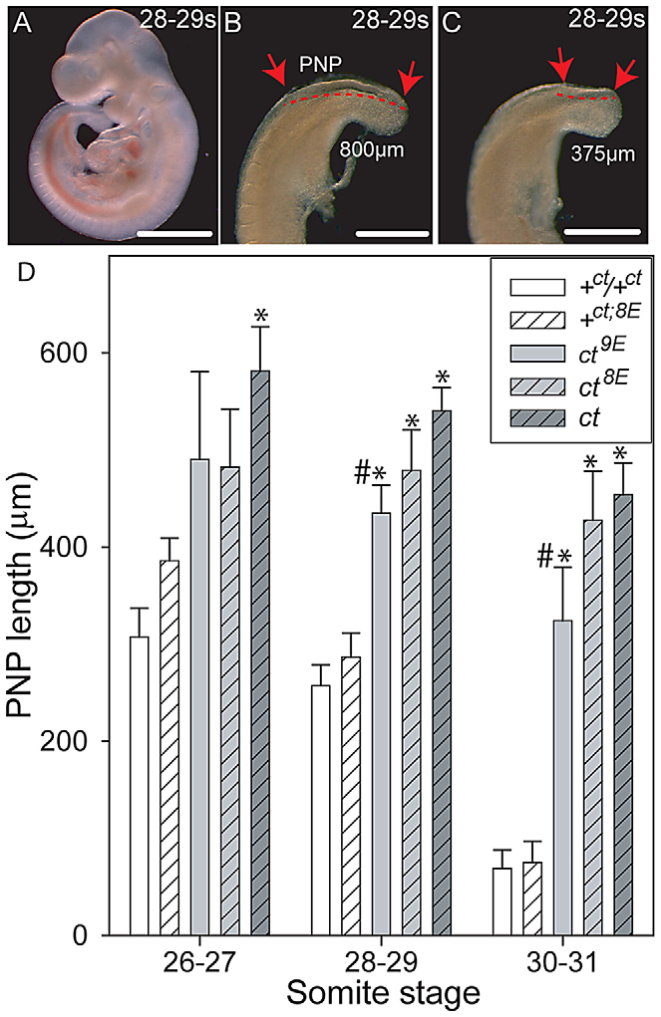
Variation in mean PNP length of embryos of the *curly tail* sub-strains. (A–C) *Curly tail* embryos at the 28–29 somite stage (E10.5), showing whole embryo (A) and enlarged views of two different caudal regions (B, C) to illustrate variable PNP lengths (between the arrows and indicated by dotted lines). An enlarged PNP (e.g. 800 µm length in B) is indicative of delay or failure of closure compared with a smaller PNP (e.g. 375 µm in C). Scale bars represent 1 mm in A and 0.5 mm in B, C. (D) PNP length measurements (mean in µm ± SEM) at somite stages around the time of PNP closure. At 28–29 and 30–31 somites, the mean PNP lengths of embryos of the *ct*, *ct^8E^* and *ct^9E^* strains (i.e. *Grhl3* mutants; grey bars) are significantly larger than those of stage-matched embryos wild-type at the *Grhl3* locus (i.e. +*^ct^* and +*^ct;8E^*; white bars), regardless of *Lmnb1* genotype (* significantly different from *Grhl3* wild-type strains, *p*<0.001; ANOVA and Holm-Sidak pairwise comparisons). This difference is also observed for the *ct* strain at the 26–27 somite stage. At each stage, the mean PNP length of +*^ct8E^* embryos is larger than for +*^ct^* embryos, but this difference does not reach statistical significance. Among *ct* sub-strains, embryos carrying the *Lmnb1^9E^* allele had the smallest PNP lengths at each stage analysed (# significantly different from *ct* strain; *p*<0.001). Number of embryos corresponding to each bar: n = 5–16 at the 26–27 somite stage; n = 14–66 at the 28–29 and 30–31 somite stages.

Although *curly tail* NTDs are partially penetrant, affected embryos can be recognised on the basis of an enlarged PNP at E10.5 [40]. In order to examine the effect of *Lmnb1* variants on the progress of spinal neural tube closure directly, PNP length was measured in a series of embryos at E10.5 (Figure 4). Among embryos that were wild-type at the *Grhl3* locus (*+^ct^* and +*^ct;8E^*), PNP length diminished rapidly between the 26 and 31 somite stages and, by the 30–31 somite stage, the PNP was very small (12 out of 37 embryos) or closed (25 of 37 embryos). There was no detectable difference between embryos with *Lmnb1^8E/8E^* and *Lmnb1^9E/9E^* genotypes. In contrast, mean PNP lengths were significantly larger in the *Grhl3^ct/ct^* sub-strains, reflecting an overall delay in closure. Although mean PNP length did not differ between *curly tail* and the *ct^8E^* sub-strain, embryos of the *ct^9E^* sub-strain exhibited a more rapid reduction in PNP length from the 28–29 somite stage onwards (Figure 4), indicative of an overall normalisation of spinal neural tube closure. The distribution of PNP lengths in embryos of the *ct^9E^* sub-strain was shifted towards smaller values, with a significantly lower mean PNP length. Moreover, only a few *ct^9E^* embryos showed very large PNPs, whereas a greater proportion of embryos had completed PNP closure by the 30–31 somite stage (8 of 30 compared with 1 out of 20 among the *ct^8E^* sub-strain; *p*<0.05, z-test; Figure S3). These observations on PNP length correlate with the diminished frequency of spina bifida in the *ct^9E^* sub-strain later in development.

Generation of the *curly tail* sub-strains provided an opportunity to test directly whether the variation in number of Glu residues is responsible for the 2-DE migration difference of lamin B1 in *curly tail* and wild-type samples. 2D gels were generated from mouse strains expressing the Lmnb1^8E^ (*ct*, *ct^8E^*, +*^8E^*) or Lmnb1^9E^ (+*^ct^*, *ct^9E^*) variants. In each case the migration pattern corresponded with the number of Glu residues (Figure S4), confirming that the characteristic strain-dependent 2-DE pattern reflects the *Lmnb1* polymorphism.

**Figure 5:**
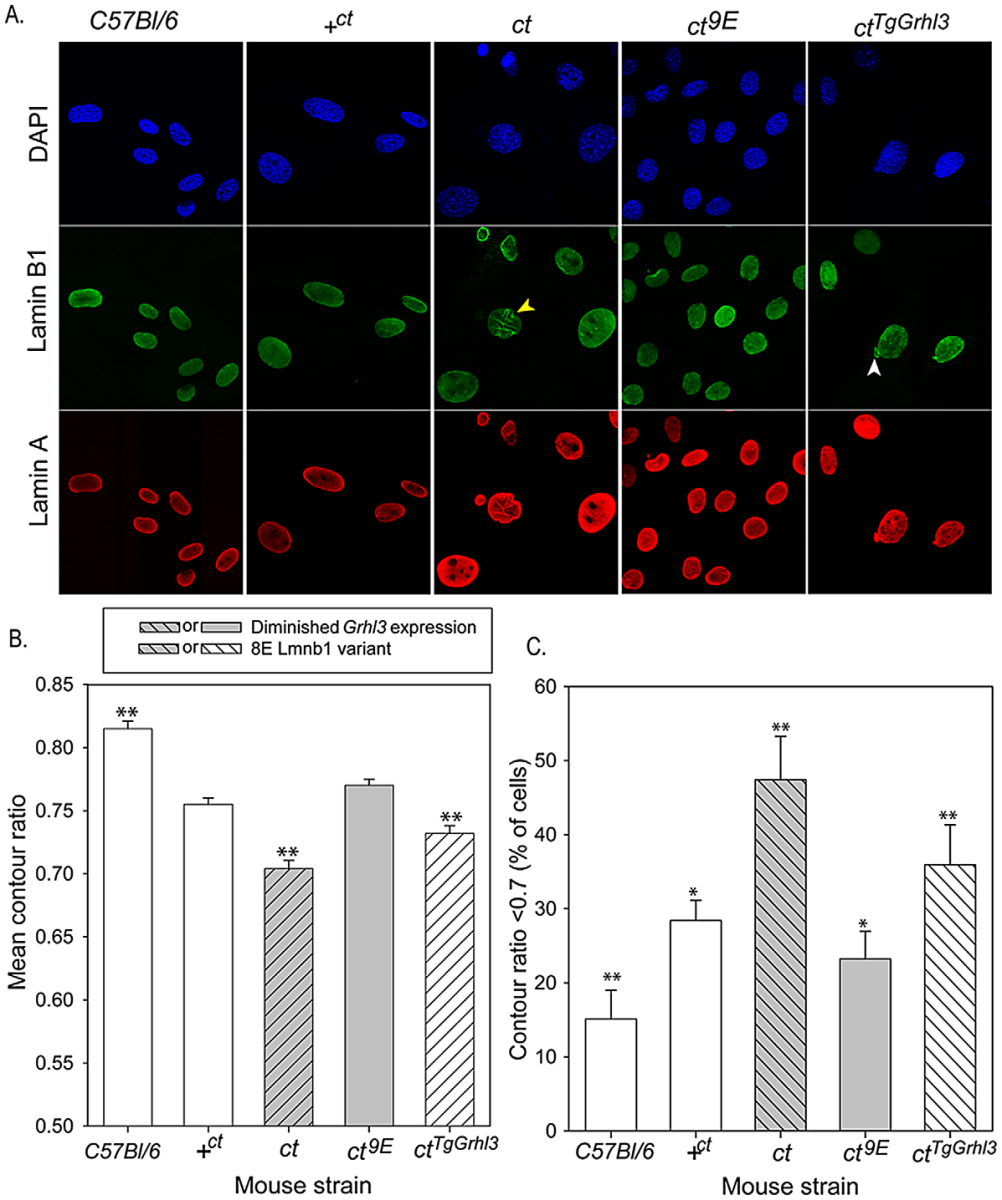
Nuclear morphology is influenced by Lamin B1 variation. (A) MEFs derived from embryos of various genotypes were stained with DAPI (blue) and antibodies to lamin B1 (green) and lamin A (red) to highlight the nuclear lamina. Abnormalities observed include lobulations (yellow arrowhead) and herniations (white arrowhead). (B, C) Analysis of MEF nuclei reveals significant differences between sub-strains in (B) mean contour ratio and (C) percentage of nuclei with contour ratio lower than 0.7, which is considered dysmorphic (*p*<0.001; ANOVA). (B) Mean contour ratio (expressed as mean ± SEM) is significantly higher in C57BL/6 and significantly lower in *ct* than all other strains. (C) Compared with all other strains, *ct* has a significantly higher frequency of dysmorphic nuclei (47.4±5.8%) and C57BL/6 (15.1±3.9%) has significantly fewer dysmorphic nuclei (** significant difference from all other strains, *p*<0.01). The proportion of dysmorphic nuclei is lower in strains with the wild-type *Lmnb1^9E^* allele, but higher than in C57BL/6; * indicates significantly different from all other strains (*p*<0.01 for comparison with C57Bl/6, *ct* and *ct^TgGrhl3^* and *p*<0.05 for comparison with *+^ct^* or *ct^9E^*). Over-expression of *Grhl3* partially normalises nuclear phenotype in the *ct^TgGrhl3^* strain (despite presence of *Lmnb1^8E^* variant as in *ct*). The mean contour ratio is significantly higher than in the *ct* strain but lower than in *ct^9E^* or +*^ct^* strains (** indicates significant difference compared with all other strains tested, *p*<0.01). Values are an average of 9–15 experiments, using 2–3 independent cell lines for each strain. Total number of cells analysed: 579 C57BL/6; 895 +*^ct^*; 757 *ct*; 882 *ct^9E^*; 837 *ct^TgGrhl3^*.

We hypothesised that the mechanism by which variation in lamin B1 sequence affects risk of NTDs could relate to the apparent effect on stability of the nuclear lamina, as shown by FLIP. In order to further examine the effects of the lamin B1 variants, we examined nuclear morphology in MEFs derived from embryos of differing strains. We previously showed that *Grhl3* is expressed in MEFs and that the expression deficit is observed in cells derived from *curly tail* embryos, as in the embryos themselves [41]. Immunostaining for lamin A and lamin B1 allowed visualisation of nuclear shape and, among *curly tail* MEFs, many nuclei showed a high degree of irregularity in shape, including herniations and/or lobulations (Figure 5A). Moreover, in a proportion of *curly tail* cells, lamin B1 staining was discontinuous. Lamin A showed a similar distribution to lamin B1, suggesting that the variant lamin B1 imposes a dysmorphic phenotype on the nuclear lamina as a whole. Abnormalities were much less frequent in nuclei of the +*^ct;9E^* and C57BL/6 strains, carrying wild-type alleles of *Lmnb1* and *Grhl3*. To provide a quantitative measure of nuclear morphology, the contour ratio (4???×area/perimeter) of DAPI-stained nuclei was analysed (Figure 5B, 5C). The mean contour ratio was significantly lower for *ct* nuclei than for any of the other strains (Figure 5B). Consistent with these findings, compared with other strains examined, a significantly greater proportion of *ct* nuclei showed a contour ratio of less than 0.7 (Figure 5C), which is considered abnormal [42].

The *curly tail* nuclear dysmorphology phenotype was rescued by the presence of the Lmnb1^9E^ variant in the *ct^9E^* sub-strain (Figure 5), correlating with the apparent increased stability of the lamina when this variant is present, as observed by FLIP (Figure 2C). Interestingly, in MEFs from a transgenic *ct* strain, *ct^TgGrhl3^* in which *Grhl3* expression is reinstated by over-expression from a *Grhl3*-containing BAC [9], the nuclear morphology was intermediate between that of *ct* and *ct^9E^* MEFs (Figure 5). Thus, although *ct^TgGrhl3^* mice are on an identical genetic background to *ct*, including the *Lmnb1^8E^* variant, it appears that over-expression of *Grhl3* is sufficient to partially ameliorate the nuclear dysmorphology phenotype. The mean contour ratio of nuclei was higher, and the proportion of abnormal nuclei was lower, for C57BL/6 than any of the other strains, including +*^ct;9E^* (Figure 5). Thus, in addition to lamin B1 sequence and *Grhl3* expression, other factors associated with the *curly tail* genetic background may influence nuclear morphology. Overall, among strains with the *curly tail* genetic background, those that express the Lmnb1^9E^ variant (+*^ct;9E^* and *ct^9E^*) have a significantly higher mean contour ratio (Figure 5B) and fewer dysmorphic nuclei (Figure 5C) than those that express the Lmnb1^8E^ variant (*ct* and *ct^TgGrhl3^*).

**Figure 6:**
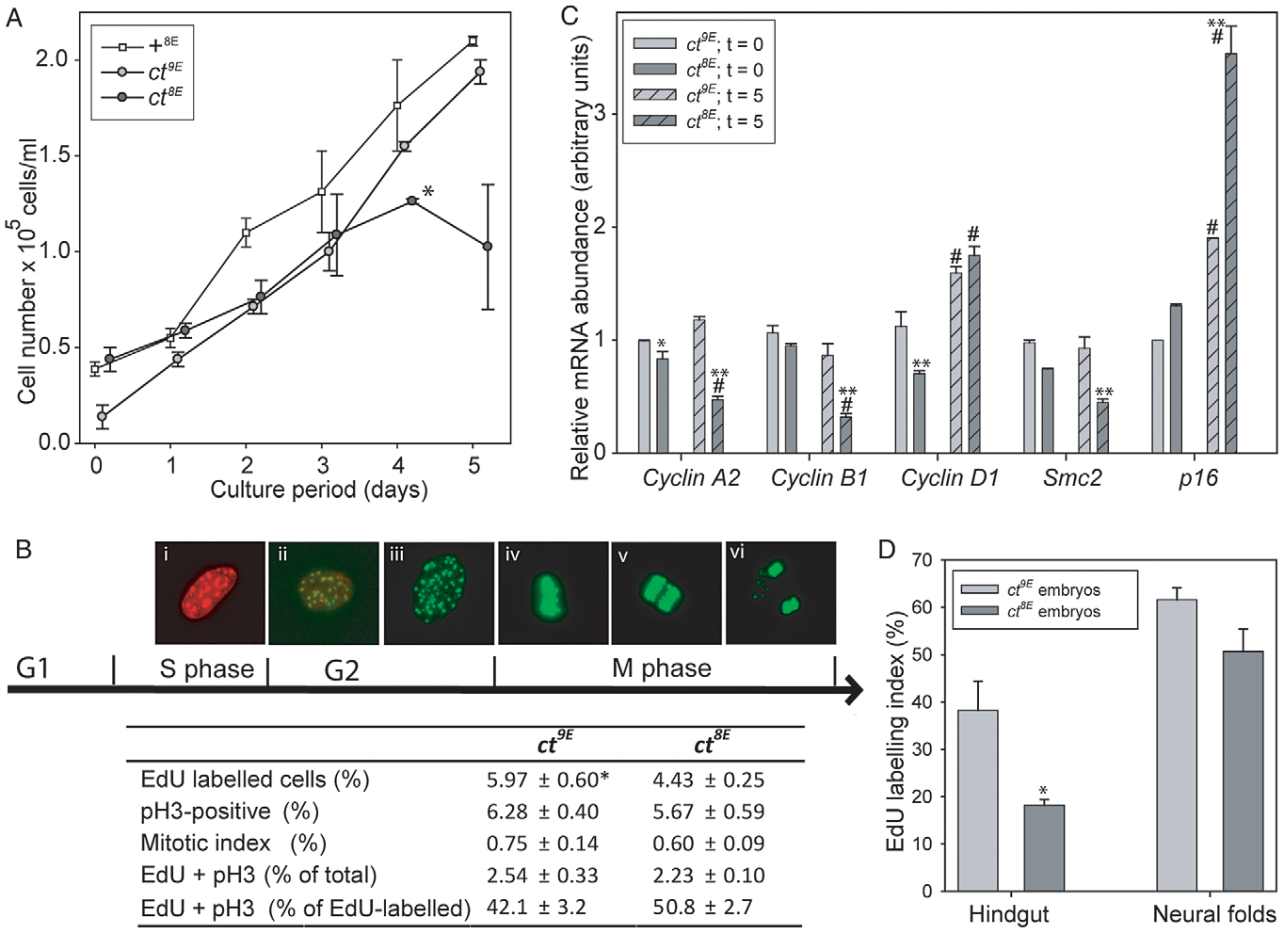
Cell cycle progression is impaired in *curly tail* cells and embryos expressing the Lmnb1^8E^ variant. In cultured embryonic fibroblasts (A–C), analysis of growth curves (A) shows that *ct^8E^* cells proliferate significantly slower than *ct^9E^* cells (*p<0.05; multiple linear regression for days 0–4) and then undergo a ‘growth crisis’ after 4 days culture. (B) Cell cycle analysis was performed at day 0 (5 hours of culture) using EdU to label cells as they progress through S phase (Bi) and anti-phospho Histone H3 (pH 3) to label cells in G2/M (Bii–vi). Mitotis was scored visually as cells that were in prophase, metaphase (Biv), anaphase (Bv) or telophase (Bvi). Data represents the mean of three experiments, each using an independent cell line, plated in triplicate. *ct^8E^* cells show significantly reduced EdU labelling (* *p*<0.05; t-test). There is a trend towards reduced pH 3 labelling and mitotic index in *ct^8E^* cells, but this difference is not statistically significant. The proportion of cells double-labelled with EdU and pH 3 (Bii) does not differ between *ct^8E^* and *ct^9E^* cells. (C) Expression of cell cycle regulators determined by qRT-PCR. For each gene, significant differences in the comparison of *ct^8E^* and *ct^9E^* cells cultured for the same period are indicated (* *p*<0.05, ** *p*<0.01; ANOVA with Holm-Sidak Pairwise Comparison). Expression differences between 0 and 5 days in culture for cells of the same genotype are indicated (# *p*<0.05). (D) Analysis of proliferation in embryos at E10.5 showed that EdU labeling index was significantly diminished in the hindgut of *ct^8E^* compared with *ct^9E^* embryos (* p<0.02; t-test).

The effect of the Lmnb1^8E^ variant on nuclear morphology and the known function of lamins in nuclear function, including DNA replication [24], prompted us to investigate the effect of the Glu variant on proliferative capacity in *ct* cells. MEFs were plated and counted after 4 hours (t = 0) and after successive 24 hour periods up to 5 days. Growth curves showed that *ct^8E^* MEFs proliferate significantly more slowly than their *ct^9E^* counterparts over the first four days in culture (*p*<0.05; Multiple linear regression, R^2^ = 0.948) and then undergo a ‘proliferative crisis’ where cell numbers cease to increase (Figure 6A). The experiment was performed on three separate occasions using independent cell lines, with the same result each time. Therefore, in addition to nuclear dysmorphology, the Lmnb1^8E^ variant is associated with an apparent reduction in proliferative capacity in *ct* cells. In contrast, *ct^9E^* cells continued to proliferate at a similar rate to wild-type +*^8E^* cells at day 5 (Figure 6A). In accordance with the growth curve data, we also noted that when MEFs were repeatedly passaged, *ct^8E^* fibroblasts show a dramatic loss of proliferative capacity from passage 5 onwards, whereas *ct^9E^* continue to exhibit similar doubling times up to at least passage 8.

To further investigate cell cycle properties of *ct^8E^* and *ct^9E^* cells, labelling with 5-ethynyl-2′-deoxyuridine (EdU; to monitor S-phase progression) and immunostaining for phospho-histone H3 (pH 3; a marker of mitosis) were performed on day 0. This is well before the profound loss of proliferative capacity that occurs in *ct^8E^* cells after extended culture and it was therefore predicted that differences, if present, may be subtle. However, corresponding with growth curve data, we observed significantly fewer EdU-labelled *ct^8E^* cells than *ct^9E^* cells (Figure 6B), together with a non-significant reduction in pH 3 labelling (cells in G2/M phase) and mitotic index. Consistent with the reduced EdU labelling in *ct^8E^* cells, indicating that fewer cells had passed through S-phase, we observed a slightly lower proportion of EdU/pH 3 double-labelled nuclei. However, there was no difference between strains in the number of double-labelled cells as a proportion of the total number of EdU-labelled cells (Figure 6B), suggesting that progression from S-phase to G2 is not defective in *ct^8E^* cells.

We next examined the expression of key regulators of cell cycle expression by qRT-PCR, 4 hours after plating (t = 0, as for cell cycle analysis) and after 5 days of culture (t = 5). The reduced proliferative capacity of *ct^8E^* cells during the initial growth period was associated with significantly lower expression of *Ccnd1*, encoding cyclin D1 (Figure 6C). After 5 days, the expression of *Ccna2* and *Ccnb1* (encoding cyclin A2 and cyclin B1, respectively) was also significantly reduced in *ct^8E^* compared with *ct^9E^* cells, consistent with diminished cell cycle progression [43], [44]. Conversely, there was a dramatic increase in expression of *p16^Ink4a^* (Figure 6C), which suppresses cell cycle progression through inhibition of cyclin D-dependent kinases [45] and is a hallmark of cells entering senescence. The expression of *p16^Ink4a^* was also increased in *ct^9E^* cells at t = 5 compared with t = 0, but to a much lesser extent. In addition, at both stages *ct^8E^* cells also exhibited a significant reduction in expression of *Smc2*, which encodes a core component of the condensin I and II complexes that play key roles in chromosome condensation during mitosis [46], [47]. We conclude that changes in expression of cell cycle-associated proteins are consistent with reduced cell cycle progression in cells expressing the Lmnb1^8E^ variant, compared with those expressing the wild-type Lmnb1^9E^ variant.

Finally, we tested whether the Lmnb1 variants were also associated with differences in cellular proliferation rate in the developing embryo. Analysis was performed on the neural folds and hindgut at the axial level of the closing PNP, at the stage at which the underlying defect in proliferation in the hindgut of affected *curly tail* embryos was reported [12], [13]. Consistent with the findings in cultured cells, the EdU labelling index was lower in *ct^8E^* than in *ct^9E^* embryos, particularly in the hindgut (Figure 6D, Table S3). Mitotic index was similar in the sub-strains (Table S3). The diminished S-phase progression of cells in the hindgut of *ct^8E^* embryos corresponds with the proliferation defect that is known to underlie spinal NTDs in *curly tail* embryos.

## Discussion

The multifactorial, partially penetrant genetics of the *curly tail* mouse provided an opportunity to investigate the *Lmnb1* polymorphism as a potential modifier of susceptibility to NTDs. In the context of the genetic background of the *curly tail* mouse, we observed a major effect of lamin B1 on development of the neural tube, the embryonic precursor of the brain and spinal cord. *Curly tail* sub-strains expressing the *Lmnb1^8E^* variant demonstrate failure of neural tube closure with significantly higher frequency than those that express wild-type protein. Thus, although both the *curly tail* sub-strains (*ct^9E^* and *ct^8E^*) are homozygous for the *Grhl3^ct^* mutation, which results in diminished *Grhl3* expression [9], there is a three-fold difference in the frequency of NTDs depending on the co-existing *Lmnb1* genotype. Strikingly, although exencephaly occurs at much lower frequency than spina bifida, *Lmnb1* also affected the penetrance of these defects to a similar extent as spinal NTDs, with approximately 65% reduction in frequency among *ct^9E^* compared with *ct^8E^* embryos. Interestingly, it appears that the Lmnb1^8E^ variant may confer susceptibility to NTDs even in the absence of a *Grhl3* mutation, at least in the context of the *ct* genetic background. Thus, +*^ct;8E^* embryos that are wild-type for *Grhl3* but which carry the *Lmnb1^8E^* variant developed occasional tail flexion defects and/or exencephaly. In contrast, spinal NTDs can be prevented by transgenic over-expression of *Grhl3* expression (*ct^TgGrhl3^*) [9], despite the presence of the *Lmnb1^8E^* variant.

The possible functional effect of polymorphic variants has been explored in very few proteins, to date. We found that the loss of a single Glu, in Lmnb1^8E^, compromises the stability of lamin B1's interaction within the nuclear lamina. This effect is predicted to result from disturbance of the conformation of the C-terminal region of the protein, owing to the location of the variant Glu residue in a predicted alpha-helix.

The effect of the Glu repeat polymorphism on lamina stability in FLIP analysis correlates with the observation of a higher proportion of dysmorphic nuclei in *ct* MEFs that express the Lmnb1^8E^ variant compared with Lmnb1^9E^. Abnormalities in lamin immunostaining and nuclear shape are reminiscent of cells with nuclear envelope abnormalities, such as from progeria models [42], lamin B1 mutant mice [33], [34] and following shRNA-mediated silencing of lamin B1 [48]. Using contour ratio analysis, abnormal nuclei were observed in around 47% of primary embryonic *curly tail* fibroblasts (current study), compared with 68% of primary dermal fibroblasts derived from a patient with Hutchison-Gilford progeria syndrome [42]. Only 7–15% of nuclei among control fibroblasts exhibited such abnormalities. In these previously reported examples, abnormalities of cell proliferation, chromosome position, transcription factor localisation and gene expression have all been noted [22], [26], [33].

We found a strong correlation between frequency of dysmorphic nuclei in MEFs derived from embryos of the *ct* strain, and frequency of NTDs. For example, among mice homozygous for the *Grhl3^ct^* hypomorphic allele, presence of the wild-type *Lmnb1^9E^* led to a reduced NTD frequency and an increased proportion of ‘normalised’ nuclei in MEFs. These findings suggest that nuclear lamina function plays a contributory role to the efficiency of neural tube closure during embryogenesis. Whether altered nuclear structure directly affects NTD risk or is a secondary marker of altered lamin B1 function is not known. To investigate the cellular mechanism by which lamin B1 affects embryonic development we focussed on a possible effect on cell cycle progression, in view of the known tissue-specific cell cycle defect that underlies spinal NTDs in *curly tail* mice [8].

Lamin B1 functions in nuclear envelope breakdown/assembly and mitotic spindle formation [23], [25], [49]. In addition, lamin B types are spatially associated with and required for DNA synthesis during S-phase [50]. Effects of lamin B1 dysfunction on cell cycle regulation could also be mediated through altered regulation of gene expression. For example, sequestration of the transcription factor Oct-1 at the nuclear periphery is lost in cells expressing a truncated form of lamin B1, resulting in mis-expression of target genes, including cell cycle mediators [27], [51]. In *ct* fibroblasts expressing the Lmnb1^8E^ variant, analysis of growth curves and cell cycle markers revealed diminished proliferative capacity and premature senescence, accompanied by characteristic changes in expression of cell cycle mediators. Cell labelling experiments suggest that the reduced proliferation rate of *ct^8E^* cells does not result from a defect at the S-phase/G2 transition but more likely from impairment of G1 or G1/S transition. Such an idea is consistent with the reduced expression of cyclin D1, which promotes progression through G1/S. The proliferative crisis that occurs in *ct^8E^* following extended culture is accompanied by reduced expression of cyclins A2 and B1, which function at G2/M [52], and increased expression of p16^Ink4a^. Although our study addresses an amino acid change rather than reduced expression, these observations are consistent with recent studies showing that silencing of *Lmnb1* expression reduces proliferation rate and induces premature senescence in fibroblast cell lines [53]. Altered cell cycle exit is also thought to be responsible for reduced thickness of the cortex in *lmnb1/lmnb2* knockout embryos [29], [34].

Rather than a generalised growth retarding effect of the lamin B1 variant, it appears that there is an additive effect with the *Grhl3^ct^* mutation. Cell cycle differences between *ct^8E^* and *ct^9E^* cells therefore suggest a mechanism by which *Lmnb1* genotype affects the morphogenetic movements of neural tube closure in *curly tail* mutant embryos. It was previously found that: (i) the cellular basis of spinal NTDs in *curly tail* mutant embryos involves a proliferation defect in cells of the hindgut which causes excessive axial curvature [12]; and (ii) inhibition of proliferation by anti-mitotics or experimental growth retardation increases frequency of cranial NTDs [18], [54]. Therefore, the reduction in cellular proliferation rate resulting from the combination of diminished *Grhl3* expression together with perturbation of lamin B1 function, would be predicted to exacerbate both spinal and cranial neurulation, as we observe. In support of this model, and correlated with prevention of NTDs in embryos, reinstatement of *Grhl3* expression in cultured cells that express the Lmnb1^8E^ variant partially normalises nuclear morphology (e.g. in *ct^TgGrhl3^*) and proliferative capacity (e.g. growth curves of *+^ct;8E^* and *ct^9E^* cells do not differ). Moreover, *in vivo* analysis confirmed that proliferation is diminished in the hindgut of *ct^8E^* compared with *ct^9E^* embryos, which suggests an explanation for their greater susceptibility to spinal NTDs.

Overall, our findings show that *Lmnb1* is a modifier gene that has a significant influence on the risk of NTDs in *curly tail* (*Grhl3^ct^*) embryos. We propose that the *Lmnb1^8E^* polymorphism and *Grhl3^ct^* mutation interact genetically to influence nuclear morphology and proliferation, and hence susceptibility to NTDs. The influence of gene-gene interactions on susceptibility to NTDs in the *curly tail* model parallels the apparent multigenic etiology of the corresponding human conditions. Thus, it appears possible that some individuals carry ‘risk’ alleles that are insufficient to cause NTDs when present in isolation, but confer susceptibility to NTDs when co-inherited with other predisposing alleles. We speculate that variation in human lamin B1, either in the Glu repeat or elsewhere in the protein, would be worthy of investigation in the context of human NTDs.

## Methods

### Maintenance of mice and genotyping

*Curly tail* (*ct/ct*), genetically-matched (partially congenic) wild-type (*+^ct^/+^ct^*) and transgenic *curly tail* mice carrying a *Grhl3-*expressing BAC (*Grhl3^ct^/Grhl3;Tg(Grhl3)1NDEG*, here referred to as *ct^TgGrhl3^*) were as described previously [8], [9]. A two-step breeding programme (Figure S2) was used to generate mice carrying different combinations of the *Grhl3* alleles (referred to as *Grhl3^+^* or *Grhl3^ct^*) and *Lmnb1* variants (referred to as *Lmnb1^9E^* and *Lmnb1^8E^*). Mice of genotype *Grhl3^ct/ct^*; *Lmnb1^8E/8E^*, *Grhl3* ^ct*/ct*^; *Lmnb1^9E/9E^* and *Grhl3^+/+^*; *Lmnb1^8E/8E^* were selected and inter-crossed to establish independent colonies.

The *Grhl3^ct^* allele was genotyped on the basis of the putative mutation, C-21350T, upstream of *Grhl3* by PCR amplification of genomic DNA with restriction digest of PCR products [9]. Genotyping was confirmed by PCR amplification of polymorphic microsatellite markers, D4Bwg1551e and D4Mit204, downstream of *Grhl3*. The *Lmnb1* GAG repeat variant (Deletion, 18: 56909394) was genotyped by PCR amplification of genomic DNA using primers that encompass the repeat (5′-GACCACCATACCCGAGGAG and 5′- TCCACAGCCACTCCGATG), with separation of products on 5% agarose gels. The C612T SNP (18: 56868078) creates a *HindIII* restriction site, allowing genotyping by PCR amplification of exon 1 (using primer pair 5′-GGCCTGTGGTTTGTACCTTC-3′ and 5′-GGCACCCCTGTTCAGTTCTA-3′), followed by restriction digest of the PCR product.

### Collection of embryos

Experimental litters were generated by timed matings. Pregnant females were killed at embryonic day by cervical dislocation and embryos were dissected from the uterus in Dulbecco's Modified Eagle's Medium (Invitrogen) containing 10% fetal calf serum (Sigma). At E10.5, the caudal regions of individual embryos at the 30–31 somite stage were excised at the level of somite 15, rinsed in phosphate buffered saline (PBS) and stored at −80°C prior to analysis by 2-DE or Western blot. For *in situ* hybridisation embryos were fixed in 4% paraformaldehyde (PFA) in PBS at 4°C overnight. Animal studies were carried out under regulations of the Animals (Scientific Procedures) Act 1986 of the UK Government, and in according with guidance issued by the Medical Research Council, UK in *Responsibility in the Use of Animals for Medical Research* (July 1993).

### Two-dimensional gel electrophoresis (2-DE)

Samples, comprising whole embryos (n = 10 of each genotype) or individual caudal regions (n = 10 of each genotype), were prepared by sonication in lysis buffer as described previously [55]. Proteins were separated by isoelectric focussing on pH gradients of pH 4–7 or 3–5.6, followed by SDS-PAGE on 12% polyacrylamide gels, as described [56]. Gels were fixed and stained using PlusOne silver stain (GE Healthcare) and scanned using a GS-800 calibrated densitometer (BioRad). Gel images were analysed using Progenesis SameSpots (Non-linear Dynamics) with separate between-genotype comparisons for whole embryos (n = 5 pH 4–7 and 5 pH 3–5.6 gels for each genotype) and caudal regions (n = 5 pH 4–7 and 5 pH 3–5.6 gels for each genotype).

### Liquid chromatography electrospray tandem mass spectrometry (LC-ESI-MS/MS)

Protein spots were excised manually from a minimum of four different gels, so that each spot was analyzed at least in quadruplicate, subjected to in-gel digestion with trypsin and analyzed by LC-ESI-MS/MS (QToF-micro; Waters Corp.) as described previously [55]. Mass spectrometry data were searched against the SwissProt database using the MASCOT search algorithm (Matrix Science, London, UK). One missed cleavage per peptide was allowed.

### Sequence analysis

Genomic DNA fragments spanning exons of *Lmnb1* were amplified by PCR (see Table S4 for primer sequences). Purified PCR products were sequenced using big dye terminator chemistry (Applied Biosystems) and analysed on a MegaBACE 1000 (Amersham). Sequence reads derived from both strands were assembled, aligned and analysed for nucleotide differences using Sequencher (GeneCodes).

### Western blot

Protein lysates (1 µg per lane) in RIPA buffer were run on 10% Bis-Tris gels (NuPage, Invitrogen) and transferred to PVDF membrane (XCell II Blot Module, Invitrogen). Immunodetection was performed by standard methodology using antibodies to lamin B1 (S-20) and β-tubulin for normalisation (primary antibodies from Santa Cruz Biotechnology and used at 1∶1000). Proteins were detected using horseradish peroxidise-conjugated secondary antibodies (DAKO), followed by development with ECL plus Western blotting detection system (GE Healthcare). Films were scanned on a GS-800 Densitometer (Bio-Rad) for quantification.

### Quantitative real–time RT–PCR

RNA was purified (TRIzol Reagent, Invitrogen) from isolated caudal regions of E10.5 embryos or from MEFs, genomic DNA removed by DNase I digestion (DNA-free, Ambion) and first strand cDNA synthesis carried out (SuperScript II, Invitrogen). qRT-PCR was performed (MESA Blue Mastermix for SYBR Assay, Eurogentec) on a 7500 Fast Real Time PCR system (Applied Biosystems), with each sample analysed in triplicate. Primers for *Lmnb1* were designed to amplify a 221 bp product (nucleotides 1267–1487 of coding sequence (Ensembl NM 010721.1; ENSMUSG00000024590). Additional primer pairs were: cyclin A2 (*Ccna2*) 5′-CATGTCACTGCTGGTCCTTC and 5′- TGATTCAAAACTGCCATCCA); cyclin B1 (*Ccnb1*) 5′-GGAAATTCTTGACAACGGTG and 5′-TGCCTTTGTCACGGCCTTAG; Cyclin D1 (*Ccnd1*) 5′-GCGTACCCTGACACCAATCT and 5′-CTCTTCGCACTTCTGCTCCT; Smc2 5′-AAATAGCCGCCCAGAAAACT and 5′-GAGCGTTCCTTGGTGTCTTC. Primers for *p16^Ink4a^* were described previously [27]. Results were normalized to *Gapdh* as described previously [9].

For microarray, RNA was further purified using the RNeasy Micro Kit (Qiagen), followed by cDNA synthesis, linear amplification and labelling of cRNA using GeneChip 3′IVT Express Kit (Affymetrix). RNA and cRNA quantity and quality were determined by Nanodrop spectrophotometer and Bioanalyser 2100 (Agilent). Affymetrix Mouse 430_2 arrays were hybridised as standard (www.affymetrix.co.uk). Files were processed in GeneSpring GX (Agilent Technologies), with application of GC-RMA normalisation and Benjamimi-Hochberg multiple testing correction.

### Whole-mount *in situ* hybridisation

Whole-mount *in situ* hybridisation, was performed as described previously [9], using a digoxygenin-labelled 561 bp cRNA probe which was complementary to nucleotides 726–1286 of the *Lmnb1* transcript/coding sequence. Embryos were embedded in gelatine-albumin and sectioned at 50 µm thickness on a vibratome.

### Photobleaching experiments

Constructs were generated in pcDNA3.1 vector by standard cloning methods, to express fusion proteins composed of a nuclear localisation signal, yellow fluorescent protein and full-length lamin B1 or C-terminal region. Plasmids were transfected into MEFs and FLIP was performed as described previously [51]. In brief, a region of interest (ROI) was photobleached at full laser power while scanning at 4% laser power elsewhere. For quantitative analysis, background intensity was subtracted, and intensities of a specific ROI outside the photobleached area were measured over time and normalized using intensities of an ROI in a transfected but non-bleached cell.

### Immunofluorescent labelling and laser-scanning confocal microscopy

MEFs, derived from pools of 3–6 embryos at E13.5, were fixed in 4% PFA in PBS for 10 min, permeabilized with 0.4% Triton X-100 in PBS for 5 min, and blocked with 0.4% fish skin gelatine in PBS for 30 min at room temperature. Incubations with primary and secondary antibodies were for 1 h each at room temperature. Primary antibodies were mouse anti-lamin B1 (8D1; [57] and rabbit anti-lamin A (ab26300: Abcam). Secondary antibodies were donkey anti–mouse and anti–rabbit (Jackson ImmunoResearch Laboratories) conjugated to Alexa Fluor 488 and Cy5 respectively. Imaging was performed using a confocal microscope (LSM 510 META; Carl Zeiss, Inc.) on an Axio Imager.Z1 (Carl Zeiss, Inc.) with a 63× NA 1.4 oil immersion objective lens. Laser lines used were 405 nm, 488 nm and 633 nm to excite DAPI, Alexa Fluor 488 and Cy5, respectively. Fluorescence was detected using the following filters: base pairs 420–480, base pairs 505–530 and long pass 650. Images were analyzed using MetaMorph (MDS Analytical Technologies) or Image Browser (Carl Zeiss, Inc.) software.

### Cell cycle analysis

MEFs were plated onto 13 mm cover slips (passage 3; 1.0×10^5^ cells per well in triplicate), cultured for 5 hours prior to addition of 10 µM EdU (Invitrogen). After 1 hour cells were fixed and processed for detection of EdU (Click-It EdU Imaging Kit). Cells were then washed in 0.1% Triton-X100 in PBS and blocked for 30 min (5% heat-inactivated goat serum, PBS-0.1% Triton, 0.15% glycine, 2 mg/ml BSA) prior to immunohistochemistry for phospho-histone H3. Primary and secondary antibodies were anti-phospho histone H3 (1∶250, Millipore) and Alexa Fluor 488-conjugated anti-rabbit (1∶500, Invitrogen). For nuclear staining, cells were incubated with Hoechst (1∶2,000 in PBS). Ten random fields were analysed per cover slip using Image J software (U.S. National Institutes of Health, Bethesda, Maryland, USA). Cells in mitosis were scored by visual inspection of pH 3-positive cells. The experiment was repeated three times, each using an independent cell line.

For *in vivo* proliferation analysis, mice were injected with 150 µg EdU at E10.5. Embryos were collected after 90 minutes, fixed in 4% PFA and processed for embedding in paraffin wax. Transverse 7 µm sections at the axial level of the closing neural folds were used for proliferation analysis (5–7 sections per embryo), as described previously [12]. Detection of EdU (Click-It EdU Imaging Kit) was followed by immunohistochemistry for phospho-histone H3 (as above) as described [13]. Fluorescent images were collected on an Axiophot microscope (Zeiss) with a DC500 camera (Leica), using FireCam software (Leica). Images were analysed using the Cell Counter plugin in Image J.

### Statistical analysis

All statistical analysis was carried out using SigmaStat (version 3.5; Systat Software Inc).

## Supporting Information

Figure S1 Expression of *Lmnb1* mRNA in *curly tail* and wild-type embryos. Whole mount *in situ* hybridisation at E10.5 shows intense expression of *Lmnb1* throughout most of the embryo, with the exception of the heart (shown at higher magnification in F–G) and dorsal surface (arrowheads in A and E). On sections (C, D, H, I; cut at the level of the dotted lines in A and E) the diminished or absent staining in the heart is also evident. Diminished expression at the dorsal surface in whole mounts appears to correspond to lack of staining in the dorsal neural tube and surface ectoderm, particularly evident in sections through the PNP region (D, I). We did not observe any consistent differences in staining pattern between strains. A sense control probe did not give signal (B). Scale bars represent 1 mm (A, B, E), 0.5 mm (F, G) or 0.1 mm (C, D, H, I). Abbreviations: H, heart; Hg, hindgut; NF, neural folds.
(TIF)

Figure S2 Breeding scheme for generation of *curly tail* sub-strains carrying different combinations of *Grhl3* and *Lmnb1* alleles. The key strains of interest were *ct^8E^* (same genotype as *ct/ct* at *Grhl3* and *Lmnb1*), and *ct^9E^* which both carry the *Grhl3* mutation, but differ in *Lmnb1* sequence. A third strain, *+^ct;8E^*, is wild-type for *Grhl3* but carries the 8E *Lmnb1* variant. The predicted frequency of each genotype is indicated. The genetic background of the +*^ct^*/+*^ct^* strain is approximately 97% *curly tail*. Therefore, following the two further backcrosses to *ct/ct* the genetic background of the resultant *ct^8E^* and *ct^9E^* sub-strains is predicted to be 99.5% *curly tail*.
(TIF)

Figure S3 Posterior neuropore length of embryos from *curly tail* sub-strains during spinal neural tube closure. The data for individual embryos is shown, with the mean PNP length (± SEM) indicated for each strain at each stage. From the 28 somite stage, a large range of values is observed, particularly within the *curly tail* and *ct^8E^* strains. Thus, at the 30–31 somite stage, PNPs ranged from closed to as much as 1 mm long, reflecting the range of possible outcomes from normal closure to spina bifida. The overall distribution of PNP lengths in embryos of the *ct^9E^* sub-strain was shifted towards smaller values.
(TIF)

Figure S4 Migration of lamin B1 protein on 2-DE correlates with number of glutamic acid residues. Two dimensional protein gels were generated using embryo samples from wild-type (+*^ct^*), *curly tail* (*ct*), *Grhl3-*BAC-transgenic *curly tail* (*ct^TgGrhl3^*) and *ct^9E^* strains. (A) Differential migration of lamin B1 (major spot arrowed) was observed in comparison of aligned gels for *ct/ct* and +*^ct^/+^ct^* samples. (B) In strains expressing the 8E lamin B1 variant (*ct* and *ct^TgGrhl3^*), alignment of lamin B1 spots was evident (software-generated spot outline is shown), whereas the corresponding spot was absent in strains expressing the Lmnb1^9E^ variant (*+^ct^* and *ct^9E^*). (C) Conversely, the major lamin B1 spot (outlined) aligned in strains expressing the Lmnb1^9E^ variant, but was absent in strains expressing the Lmnb1^8E^ variant.
(TIF)

Table S1 Identification of lamin B1 spots by LC-MS/MS. Three spots found to migrate differentially on 2-DE of *curly tail* and wild-type samples were excised from gels and subjected to liquid chromatography coupled to electrospray tandem mass spectrometry (LC-MS/MS). Spots are numbered 1–3 from basic to acidic (right to left on 2-DE images in Figure 1), with spot 1 the most abundant in each case. The identified peptides are listed with the MASCOT score and p-value for confidence of identification. For the most abundant spot on *curly tail* gels 40% coverage of the protein was achieved.
(DOC)

Table S2 Expression analysis of genes located in proximity to *Lmnb1* on chromosome 18. A gene-list was generated that corresponds to genes located in a 41 Mb interval surrounding *Lmnb1* on chromosome 18:32.47–73.80 Mb (between markers D18Mit88 and Dev1), using UCSC Genome Browser (assembly NCBI37/mm9). This list was used to interrogate a list of genes that were found by microarray analysis to be differentially expressed (p<0.05; fold-change 1.5 or greater) in *+^ct^/+^ct^* and *ct/ct* embryos (caudal region of embryos at the 28–29 somite stage). This analysis identified 11 genes, whose relative level of expression in *+^ct^/+^ct^* compared with *ct/ct* microarray samples is indicated. Expression was evaluated by qRT-PCR (n = 5 of each genotype, repeated twice). Four genes (underlined) showed the same trend in expression in both microarray and qRT-PCR analysis, two of which (indicated in bold) were also found to significantly differ in expression between *ct* and +*^ct^* (*p<0.05; **p<0.001) by qRT-PCR. *1500015A07Rik* and *Grpel2* also showed significantly altered expression on both microarray and qRT-PCR analysis but the direction of altered expression was not consistent. None of the genes showed a significant difference in expression in comparison of *ct^9E^* and *ct^8E^* samples (n = 5 of each genotype, repeated twice; NS indicates non-significant difference). NT, indicates not tested. Note: Lack of correlation between microarray and qRT-PCR for some genes may relate in several cases to low level expression of these genes, which may have given rise to a false positive on the microarray.
(DOCX)

Table S3 Analysis of cellular proliferation rate in embryos of the *ct^9E^* and *ct^8E^* sub-strains. The proportion of cells labelled with EdU (following 90 minute treatment) and the mitotic index (based visual inspection of phospho-histone H3 positive cells) was determined at the axial level of the closure point of the neural folds in *ct^9E^* (n = 6) and *ct^8E^* (n = 5) embryos at E10.5 (mean number of somites = 28.5±0.8 and 27.6±0.5, respectively). The EdU labelling index in the hindgut was significantly higher in *ct^9E^* than in *ct^8E^* embryos (* p<0.02). There was a trend towards increased EdU labelling in the neural folds of *ct^9E^* embryos, but this did not reach statistical significance (p = 0.06).
(DOCX)

Table S4 Primers for sequencing of *Lmnb1* exons. All primers are flanking coding regions of the respective exons. Sizes of product-fragments are given in base pairs (bp), including primer sequence. T° indicates annealing temperature used for PCR amplification.
(DOCX)
