# Supplementary figures and images for: Lamin B1 Polymorphism Influences Morphology of the Nuclear Envelope, Cell Cycle Progression, and Risk of Neural Tube Defects in Mice
Source: PLoS Genet. 2012 Nov 15;8(11):e1003059. doi: 10.1371/journal.pgen.1003059 (PMC3499363; doi:10.1371/journal.pgen.1003059)

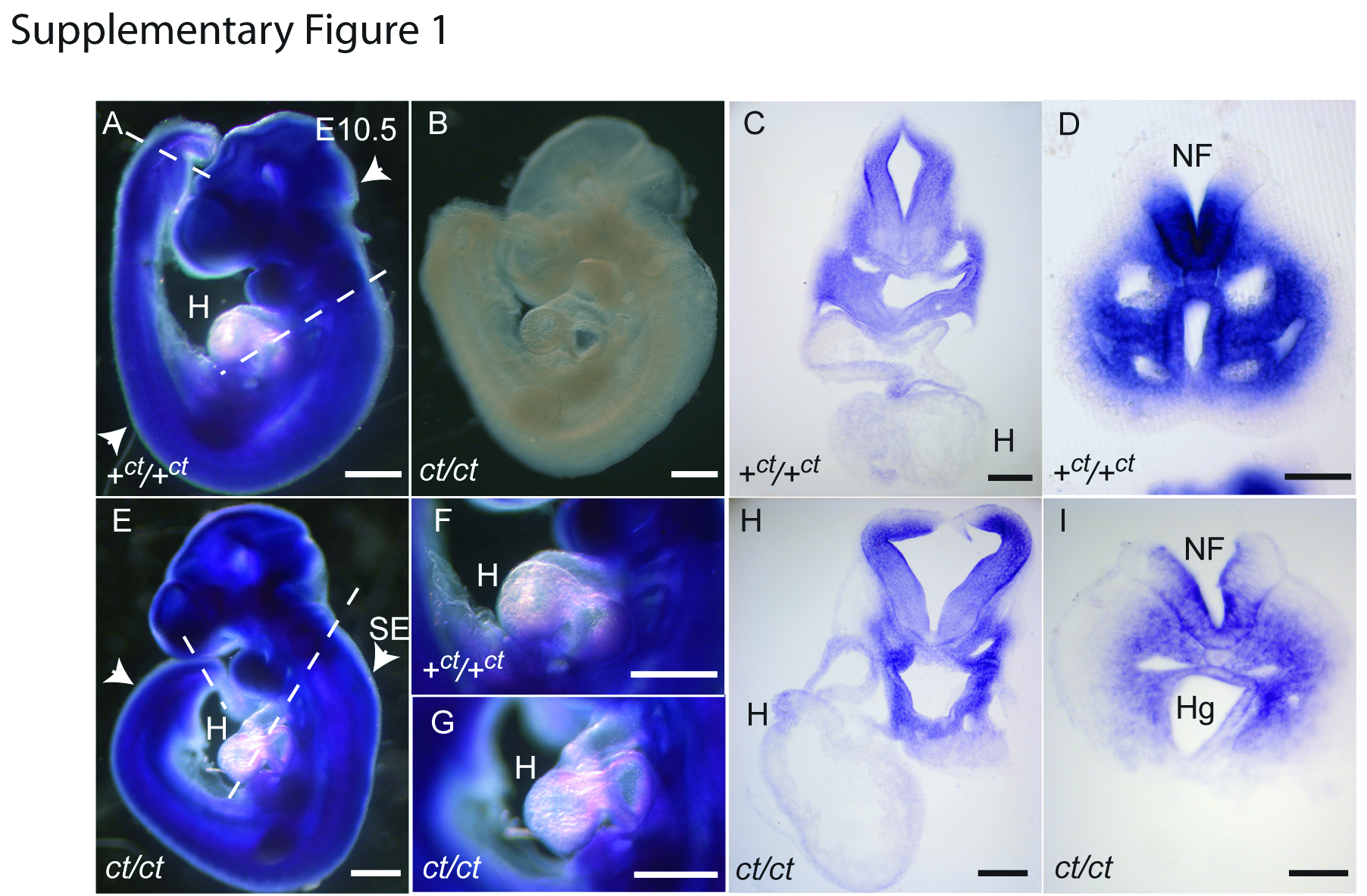

Supplement: Figure S1 — Expression of Lmnb1 mRNA in curly tail and wild-type embryos. Whole mount in situ hybridisation at E10.5 shows intense expression of Lmnb1 throughout most of the embryo, with the exception of the heart (shown at higher magnification in F–G) and dorsal surface (arrowheads in A and E). On sections (C, D, H, I; cut at the level of the dotted lines in A and E) the diminished or absent staining in the heart is also evident. Diminished expression at the dorsal surface in whole mounts appears to correspond to lack of staining in the dorsal neural tube and surface ectoderm, particularly evident in sections through the PNP region (D, I). We did not observe any consistent differences in staining pattern between strains. A sense control probe did not give signal (B). Scale bars represent 1 mm (A, B, E), 0.5 mm (F, G) or 0.1 mm (C, D, H, I). Abbreviations: H, heart; Hg, hindgut; NF, neural folds. (TIF) [file pgen.1003059.s001.tif]

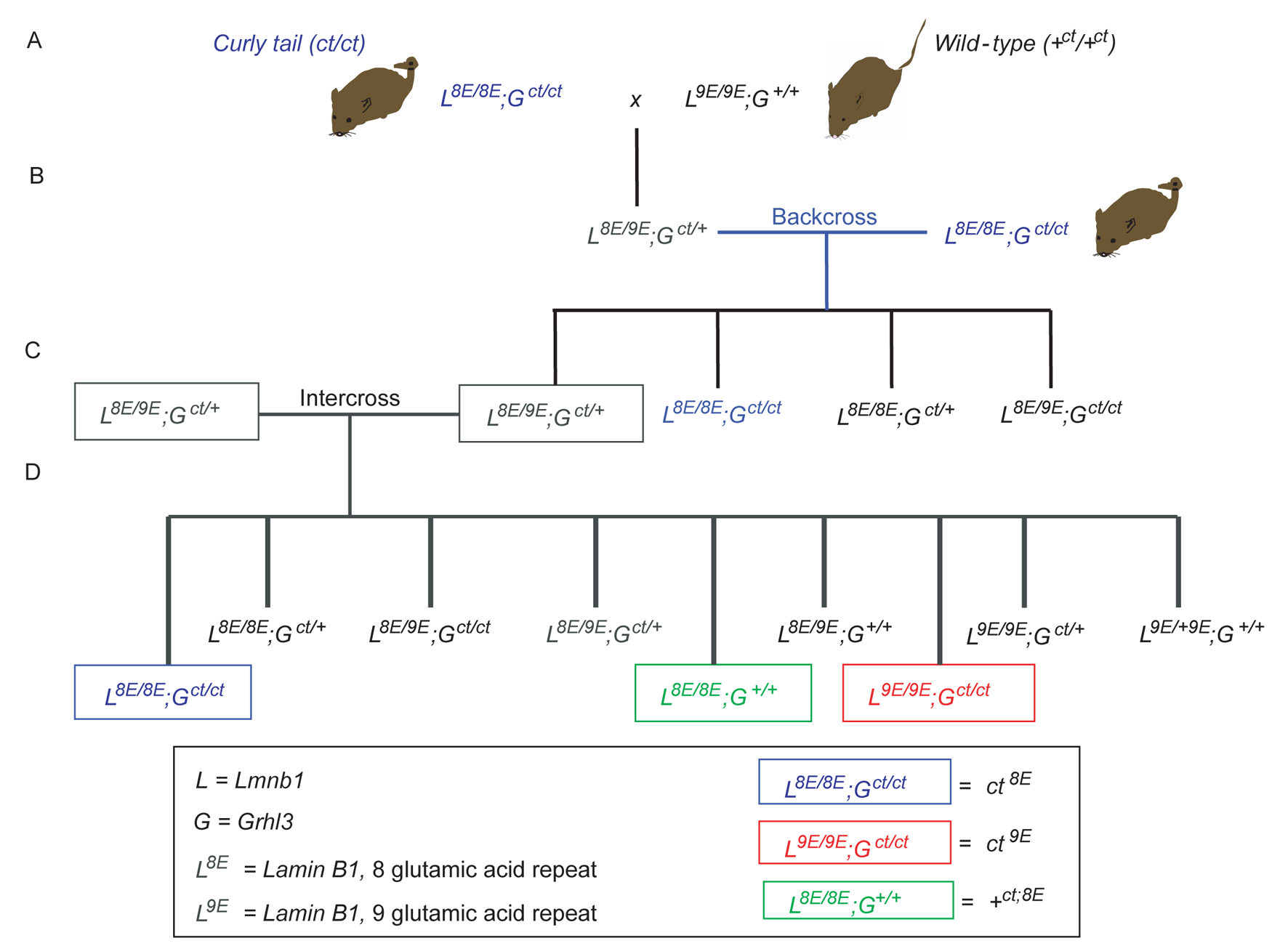

Supplement: Figure S2 — Breeding scheme for generation of curly tail sub-strains carrying different combinations of Grhl3 and Lmnb1 alleles. The key strains of interest were ct8E (same genotype as ct/ct at Grhl3 and Lmnb1), and ct9E which both carry the Grhl3 mutation, but differ in Lmnb1 sequence. A third strain, +ct;8E, is wild-type for Grhl3 but carries the 8E Lmnb1 variant. The predicted frequency of each genotype is indicated. The genetic background of the +ct/+ct strain is approximately 97% curly tail. Therefore, following the two further backcrosses to ct/ct the genetic background of the resultant ct8E and ct9E sub-strains is predicted to be 99.5% curly tail. (TIF) [file pgen.1003059.s002.tif]

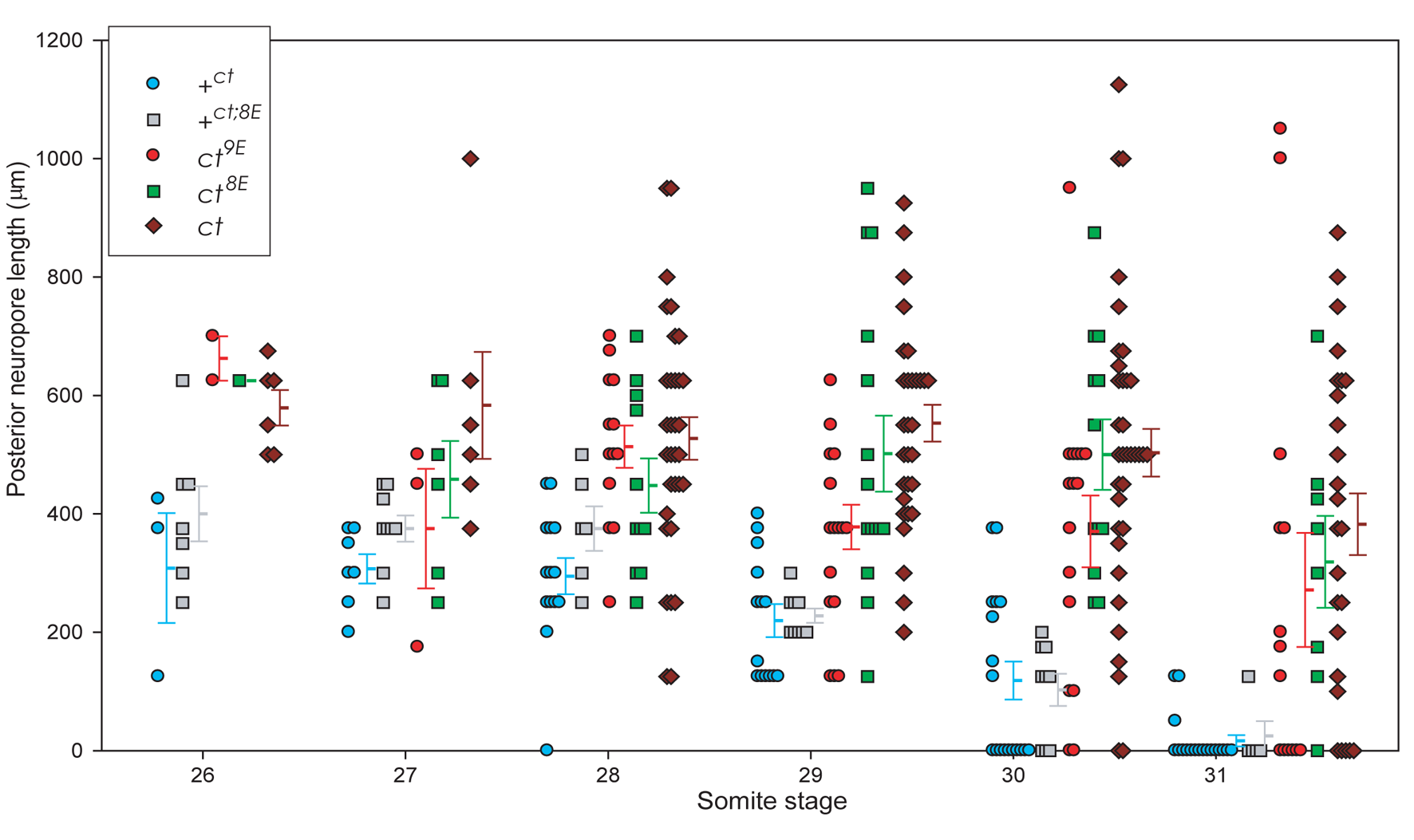

Supplement: Figure S3 — Posterior neuropore length of embryos from curly tail sub-strains during spinal neural tube closure. The data for individual embryos is shown, with the mean PNP length (± SEM) indicated for each strain at each stage. From the 28 somite stage, a large range of values is observed, particularly within the curly tail and ct8E strains. Thus, at the 30–31 somite stage, PNPs ranged from closed to as much as 1 mm long, reflecting the range of possible outcomes from normal closure to spina bifida. The overall distribution of PNP lengths in embryos of the ct9E sub-strain was shifted towards smaller values. (TIF) [file pgen.1003059.s003.tif]

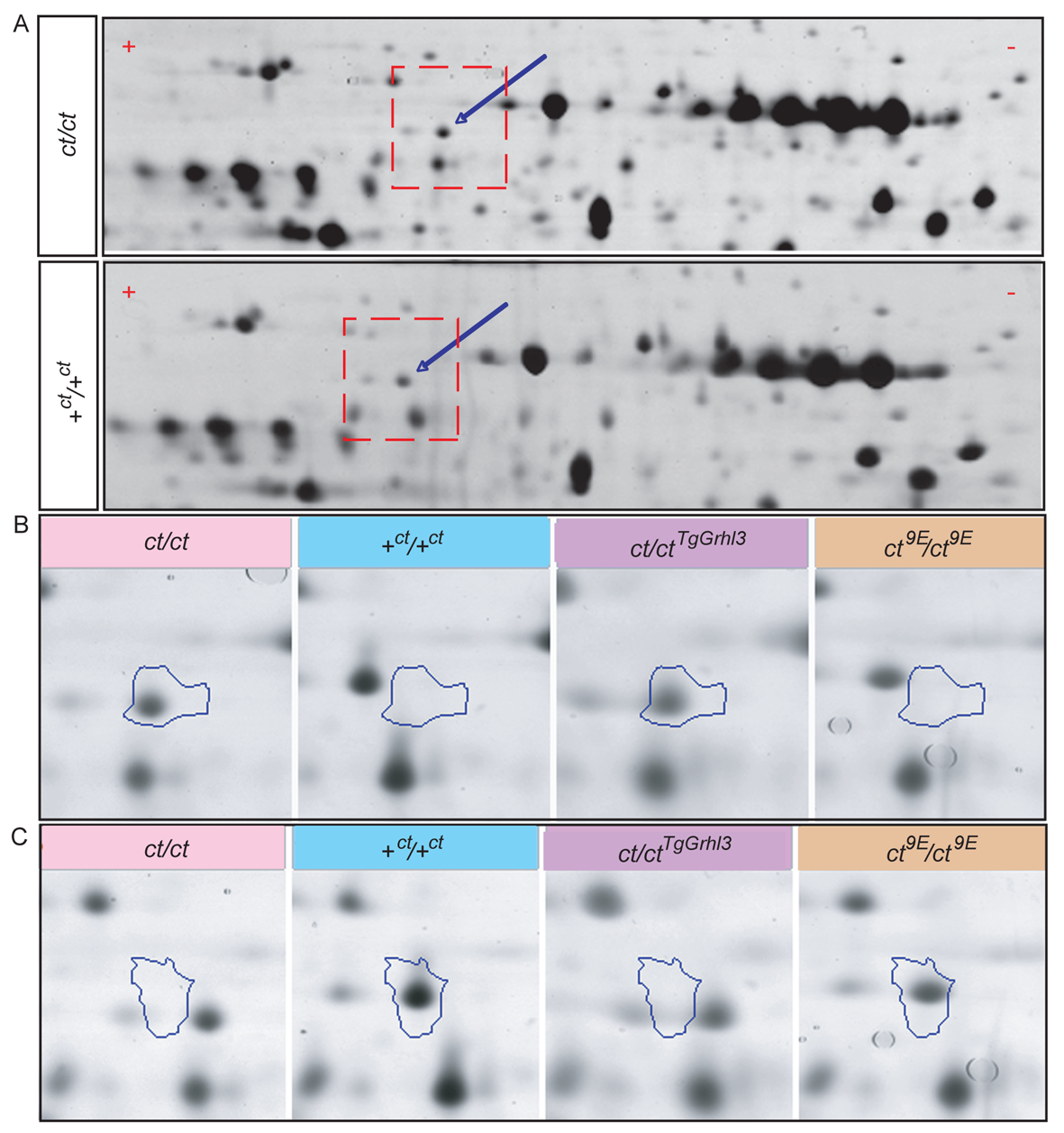

Supplement: Figure S4 — Migration of lamin B1 protein on 2-DE correlates with number of glutamic acid residues. Two dimensional protein gels were generated using embryo samples from wild-type (+ct), curly tail (ct), Grhl3-BAC-transgenic curly tail (ctTgGrhl3) and ct9E strains. (A) Differential migration of lamin B1 (major spot arrowed) was observed in comparison of aligned gels for ct/ct and +ct/+ct samples. (B) In strains expressing the 8E lamin B1 variant (ct and ctTgGrhl3), alignment of lamin B1 spots was evident (software-generated spot outline is shown), whereas the corresponding spot was absent in strains expressing the Lmnb19E variant (+ct and ct9E). (C) Conversely, the major lamin B1 spot (outlined) aligned in strains expressing the Lmnb19E variant, but was absent in strains expressing the Lmnb18E variant. (TIF) [file pgen.1003059.s004.tif]
