# Supplementary material for: Lamin B1 Polymorphism Influences Morphology of the Nuclear Envelope, Cell Cycle Progression, and Risk of Neural Tube Defects in Mice
Source: PLoS Genet. 2012 Nov 15;8(11):e1003059. doi: 10.1371/journal.pgen.1003059 (PMC3499363; doi:10.1371/journal.pgen.1003059)
Supplement: Table S1 — Identification of lamin B1 spots by LC-MS/MS. Three spots found to migrate differentially on 2-DE of curly tail and wild-type samples were excised from gels and subjected to liquid chromatography coupled to electrospray tandem mass spectrometry (LC-MS/MS). Spots are numbered 1–3 from basic to acidic (right to left on 2-DE images in Figure 1), with spot 1 the most abundant in each case. The identified peptides are listed with the MASCOT score and p-value for confidence of identification. For the most abundant spot on curly tail gels 40% coverage of the protein was achieved. (DOC) [file pgen.1003059.s005.doc]

**Supplementary Table 1**

| Strain/spot no. | | No. matched peptides | Peptide sequence (amino acid position) | Mascot score | p-value |
| --- | --- | --- | --- | --- | --- |
| *curly tail* (*ct*) | 1 | 24 | ASAPATPLSPTR(16-27)  LVEVDSGR(28-35)  LAVYIDKVR(44-50)  SLETENSALQLQVTER(53-68)  ALYETELADAR(81-91)  RALDDTAR(92-98)  AKLQIELGK(102-110)  FKAEHDQLLLNYAK(111-124)  AEHDQLLLNYAK(113-124)  ESDLSGAQIK(126-135)  LREYEAALNSK(136-146)  DAALATALGDKK(147-158)  CQSLTEDLEFR(199-209)  KNMYEEEINETR(210-221)  LAQALHEMR(243-251)  LYKEELEQTYHAK(260-272)  LSSEMNTSTVNSAR(278-291)  IESLSSQLSNLQK(301-313)  NTSEQDQPMGGWEMIR(459-474)  KIGDTSVSYK(475-484)  IGDTSVSYK(476-484)  NQNSWGTGEDVK(517-529)  NSQGEEVAQR(534-543) | 47  44  54  68  59  61  40  39  36  46  62  79  68  33  54  47  73  75  24  54  49  54  63 | 0.0007  0.045  0.00073  0.00033  0.00022  0.0017  0.013  0.0048  0.012  0.0028  0.0018  4.3e-06  5.5e-05  0.0092  0.01  0.0017  6.1e-06  4.1e-05  0.016  0.0048  0.031  0.00018  0.0019 |
|  | 2 | 8 | ASAPATPLSPTR(16-27)  SLETENSALQLQVTER(53-68)  ALYETELADAR(81-91)  RALDDTAR(92-98)  DAALATALGDKK(147-158)  SLEGDLEDLKDQIAQLEASLSAAKK(159-183)  LSSEMNTSTVNSAR(278-291)  IESLSSQLSNLQK(301-313) | 53  77  51  32  27  86  29  34 | 0.014  4.1e-05  0.025  0.046  0.051  5.3e-06  0.02  0.029 |
|  | 3 | 2 | ASAPATPLSPTR(16-27)  SLETENSALQLQVTEREEVR(53-72) | 44  21 | 0.0018  0.02 |
| Wild-type (+*^ct^*) | 1 | 13 | ASAPATPLSPTR(16-27)  LAVYIDKVR(44-50)  ALYETELADAR(81-91)  KESDLSGAQIK(125-135)  DAALATALGDKK(147-158)  LSSEMNTSTVNSAR(278-291)  IESLSSQLSNLQK(301-313)  IQELEDMLAKER(322-333)  KLLEGEEERLK(380-390)  KIGDTSVSYK(475-484)  IGDTSVSYK(476-484)  NQNSWGTGEDVK(517-529)  NSQGEEVAQR(534-543) | 63  54  68  67  64  56  62  59  49  58  68  51  62 | 0.0014  0.014  0.00051  0.00069  0.0015  0.0069  0.0022  0.0037  0.044  0.0037  0.00037  0.018  0.0025 |
|  | 2 | 4 | DQMQQQLSDYEQLLDVK(352-368)  NSQGEEVAQR(534-543)  ASAPATPLSPTR(16-27)  ALYETELADAR(81-91) | 56  49  63  52 | 0.0061  0.043  0.0015  0.022 |
|  | 3 | 1 | DAALATALGDKK(147-158) | 57 | 0.0054 |

**Table S1. Identification of lamin B1 spots by LC-MS/MS.** Three spots found to migrate differentially on 2-DE of *curly tail* and wild-type samples were excised from gels and subjected to liquid chromatography coupled to electrospray tandem mass spectrometry (LC-MS/MS). Spots are numbered 1-3 from basic to acidic (right to left on 2-DE images in Fig. 1), with spot 1 the most abundant in each case. The identified peptides are listed with the MASCOT score and p-value for confidence of identification. For the most abundant spot on *curly tail* gels 40% coverage of the protein was achieved.
