# Supplementary material for: Lamin B1 Polymorphism Influences Morphology of the Nuclear Envelope, Cell Cycle Progression, and Risk of Neural Tube Defects in Mice
Source: PLoS Genet. 2012 Nov 15;8(11):e1003059. doi: 10.1371/journal.pgen.1003059 (PMC3499363; doi:10.1371/journal.pgen.1003059)
Supplement: Table S2 — Expression analysis of genes located in proximity to Lmnb1 on chromosome 18. A gene-list was generated that corresponds to genes located in a 41 Mb interval surrounding Lmnb1 on chromosome 18:32.47–73.80 Mb (between markers D18Mit88 and Dev1), using UCSC Genome Browser (assembly NCBI37/mm9). This list was used to interrogate a list of genes that were found by microarray analysis to be differentially expressed (p<0.05; fold-change 1.5 or greater) in +ct/+ct and ct/ct embryos (caudal region of embryos at the 28–29 somite stage). This analysis identified 11 genes, whose relative level of expression in +ct/+ct compared with ct/ct microarray samples is indicated. Expression was evaluated by qRT-PCR (n = 5 of each genotype, repeated twice). Four genes (underlined) showed the same trend in expression in both microarray and qRT-PCR analysis, two of which (indicated in bold) were also found to significantly differ in expression between ct and +ct (*p<0.05; **p<0.001) by qRT-PCR. 1500015A07Rik and Grpel2 also showed significantly altered expression on both microarray and qRT-PCR analysis but the direction of altered expression was not consistent. None of the genes showed a significant difference in expression in comparison of ct9E and ct8E samples (n = 5 of each genotype, repeated twice; NS indicates non-significant difference). NT, indicates not tested. Note: Lack of correlation between microarray and qRT-PCR for some genes may relate in several cases to low level expression of these genes, which may have given rise to a false positive on the microarray. (DOCX) [file pgen.1003059.s006.docx]

| Gene name (Symbol) | Primers for qRT-PCR | Microarray fold-change  *+^ct^/+^ct^* *vs* *ct/ct* | qRT-PCR  *+^ct^/+^ct^* *vs* *ct/ct* | qRT-PCR  *ct^9E^*/*ct^9E^* *vs* *ct^8E^/ct^8E^* |
| --- | --- | --- | --- | --- |
| *Adenomatosis polyposis coli down-regulated 1* (*Apcdd1*) | 5’- AAGCAGTATCCCCACCACAG  5’- TATGGGAGGGTGGTGTTCAT | **1.54 down*** | **2.34 down**** | 1.22 down^NS^ |
| *ATPase family gene 3-like 2* (*Afg3l2*) | 5’- GGGAGAGAAATCACGTGGAA  5’- TTCCGCTCAAATGTGTCAAC | 1.74 up* | 1.10 down | NT |
| *Casein kinase 1, alpha 1* (*Csnk1a1*) | 5’- AGCATCAATGCACATCTTGG  5’- GCAGGAAACCCCTTACACAA | 2.41 down* | 1.12 down | NT |
| *GrpE-like 2*, mitochondrial (*Grpel2*) | 5’- ACCAAGCATGGCCTAGAGAA  5’- GGTTTGGTTCCAGTGACCTG | 1.89 up* | 1.26 down* | 1.31 down^NS^ |
| *Isochorismatase domain containing 1* (*Isoc1*) | 5’- TGTGGGACAGAGACTGTTGC  5’- CACGTGCGTTTCTACTCCAA | 3.5 up* | 1.17 down | 1.25 down^NS^ |
| *Matrin 3* (*Matr3*) | 5’- TGTCTTCTCAACACCGTGGA  5’- GGGCCTTCTTCAGTTCTCCT | 2.29 up* | 1.20 down | 1.06 up^NS^ |
| *RIKEN cDNA A730017C20* gene (*A730017C20Rik*) | 5’- CTGAGAGTCCTCCAGCATCC  5’- TACGTATCCCCCAACCAAAA | 5.93 up* | 1.03 up | 1.13 up^NS^ |
| [*RIKEN cDNA 1500015A07* gene](http://david.abcc.ncifcrf.gov/geneReportFull.jsp?rowids=469700) (*1500015A07Rik*) | 5’- ATGGTTTGTTTTCCGACCTG  5’- TGCTGTATCCCCAGCTCTCT | 2.77 down* | 2.64 up* | 1.17 up^NS^ |
| *Ring finger protein 14* (*Rnf14*) | 5’- CCCCACCTTCATTCACACTT  5’- GCTGTTGCCCTTCTCTGAAC | 1.78 down* | 1.00 | NT |
| *Treacher Collins Franceschetti syndrome 1*, homolog (*Tcof1*) | 5’- GATCCTCCAGCAAGAACAGC  5’- TCTGACATGGGGACCTTTTC | 1.70 up* | 1.28 down | 1.43 up^NS^ |
| *Tubulin, beta 6* (*Tubb6*) | 5’- TGAGCATTGCGACTGTCTTC  5’- TGTCGATGCAGTAGGTCTCG | **1.50 down*** | **1.30 down*** | 1.20 down^NS^ |

**Table S2. Expression analysis of genes located in proximity to *Lmnb1* on chromosome 18.** A gene-list was generated that corresponds to genes located in a 41 Mb interval surrounding *Lmnb1* on chromosome 18:32.47-73.80 Mb (between markers D18Mit88 and Dev1), using UCSC Genome Browser (assembly NCBI37/mm9). This list was used to interrogate a list of genes that were found by microarray analysis to be differentially expressed (p<0.05; fold-change 1.5 or greater) in ***+^ct^/+^ct^*** and *ct/ct* embryos (caudal region of embryos at the 28-29 somite stage). This analysis identified 11 genes, whose relative level of expression in ***+^ct^/+^ct^*** compared with *ct/ct* microarray samples is indicated. Expression was evaluated by qRT-PCR (n = 5 of each genotype, repeated twice). Four genes (underlined) showed the same trend in expression in both microarray and qRT-PCR analysis, two of which (indicated in bold) were also found to significantly differ in expression between *ct* and +*^ct^* (*p<0.05; **p<0.001) by qRT-PCR. *1500015A07Rik* and *Grpel2* also showed significantly altered expression on both microarray and qRT-PCR analysis but the direction of altered expression was not consistent. None of the genes showed a significant difference in expression in comparison of *ct^9E^* and *ct^8E^* samples (n = 5 of each genotype, repeated twice; NS indicates non-significant difference). NT, indicates not tested.

Note: Lack of correlation between microarray and qRT-PCR for some genes may relate in several cases to low level expression of these genes, which may have given rise to a false positive on the microarray.
