# Supplementary material for: Lamin B1 Polymorphism Influences Morphology of the Nuclear Envelope, Cell Cycle Progression, and Risk of Neural Tube Defects in Mice
Source: PLoS Genet. 2012 Nov 15;8(11):e1003059. doi: 10.1371/journal.pgen.1003059 (PMC3499363; doi:10.1371/journal.pgen.1003059)
Supplement: Table S3 — Analysis of cellular proliferation rate in embryos of the ct9E and ct8E sub-strains. The proportion of cells labelled with EdU (following 90 minute treatment) and the mitotic index (based visual inspection of phospho-histone H3 positive cells) was determined at the axial level of the closure point of the neural folds in ct9E (n = 6) and ct8E (n = 5) embryos at E10.5 (mean number of somites = 28.5±0.8 and 27.6±0.5, respectively). The EdU labelling index in the hindgut was significantly higher in ct9E than in ct8E embryos (* p<0.02). There was a trend towards increased EdU labelling in the neural folds of ct9E embryos, but this did not reach statistical significance (p = 0.06). (DOCX) [file pgen.1003059.s007.docx]

|  |  | **ct^9E^** | *ct^8E^* |
| --- | --- | --- | --- |
| **Hindgut** | Cells analysed | 1268 | 1772 |
|  | EdU labelling (%) | 38.2 ± 6.2 * | 18.2 ± 1.2 |
|  | Mitotic index (%) | 2.5 ± 0.3 | 2.7 ± 0.4 |
|  |  |  |  |
| **Neural folds** | Cells analysed | 3704 | 4315 |
|  | EdU labelling (%) | 61.6 ± 2.5 | 50.7 ± 4.7 |
|  | Mitotic index (%) | 4.2 ± 0.5 | 3.5 ± 0.5 |

**Table S3. Analysis of cellular proliferation rate in embryos of the *ct^9E^* and *ct^8E^* sub-strains.** The proportion of cells labelled with EdU (following 90 minute treatment) and the mitotic index (based visual inspection of phospho-histone H3 positive cells) was determined at the axial level of the closure point of the neural folds in *ct^9E^* (n = 6) and *ct^8E^* (n = 5) embryos at E10.5 (mean number of somites = 28.5 ± 0.8 and 27.6 ± 0.5, respectively). The EdU labelling index in the hindgut was significantly higher in *ct^9E^* than in *ct^8E^* embryos (* p < 0.02). There was a trend towards increased EdU labelling in the neural folds of *ct^9E^* embryos, but this did not reach statistical significance (p = 0.06).
