# Supplementary material for: Lamin B1 Polymorphism Influences Morphology of the Nuclear Envelope, Cell Cycle Progression, and Risk of Neural Tube Defects in Mice
Source: PLoS Genet. 2012 Nov 15;8(11):e1003059. doi: 10.1371/journal.pgen.1003059 (PMC3499363; doi:10.1371/journal.pgen.1003059)
Supplement: Table S4 — Primers for sequencing of Lmnb1 exons. All primers are flanking coding regions of the respective exons. Sizes of product-fragments are given in base pairs (bp), including primer sequence. T° indicates annealing temperature used for PCR amplification. (DOCX) [file pgen.1003059.s008.docx]

| **Primer name** | **Primer sequence** | **Size and T**˚ |
| --- | --- | --- |
| LmnB1_F1ex1  LmnB1_R1in1 | 5’-AGCCCGAGAGGAAACAAAGT-3’  5’-CTGCGCACCTTATCGATGTA-3’ | 411 bp  55˚C |
| LmnB1_F1in2  LmnB1_R1ex2 | 5’-GGCCTGTGGTTTGTACCTTC-3’  5’-GGCACCCCTGTTCAGTTCTA-3’ | 568 bp  55˚C |
| Lmnb1_F1up  Lmnb1_R1up | 5’-CGCTCCCGTTTACTTTTGAA-3’  5’-GTACACGCACACTCGCAGAC-3’ | 551 bp  55˚C |
| LmnB1_F2  LmnB1_R2 | 5’-catgtgatgtggggaatgtc-3’  5’-atggtggctcacaaccatct-3’ | 482 bp  52˚C |
| LmnB1_F3  LmnB1_R3 | 5’-aacggccccaaagttaaaat-3’  5’-gaccagcctaggcaacagag-3’ | 352 bp  60˚C |
| LmnB1_F4*  LmnB1_R4* | 5’-gacaggcacagaggttggtc-3’  5’-atgcatgcagacaaaacacc-3’ | 396 bp  60/55˚C |
| LmnB1_F5  LmnB1_R5 | 5’-agcgtgctttcctgtttcat-3’  5’-tgtctcatctgggacaaaaga-3’ | 367 bp  60˚C |
| LmnB1_F6  LmnB1_R6 | 5’-aagagctagagctcctttaggg-3’  5’-agagcagtcctaaaataatggaga-3’ | 454 pb  60˚C |
| LmnB1_F7  LmnB1_R7 | 5’-acatggtgagggacctcttg-3’  5’-gcatccagaagacggatagc-3’ | 409 bp  60˚C |
| LmnB1_F8  LmnB1_R8 | 5’-tctggggtcatggatttgat-3’  5’-gcctccctcttctggagtgt-3’ | 301 bp  60˚C |
| LmnB1_F9  LmnB1_R9 | 5’-aagcagctggacagagtcgt-3’  5’-tgtggtgtgtccgttttctc-3’ | 370 bp  60˚C |
| LmnB1_F10  LmnB1_R10 | 5’-tcgaaggtctactaactgctgct-3’  5’-gaaacggtaaagggctacca-3’ | 400 bp  60˚C |
| LmnB1_F11_p1  LmnB1_R11_p1 | 5’-aggtgtgagctggctcattt-3’  5’-GGACACGCAGTGGTTTTCTT-3’ | 511 bp  60˚C |
| LmnB1_F11_p2  LmnB1_R11_p2 | 5’-CCCTCAAGTTTTTGGCATTT-3’  5’-ttaaaagggccagtcacctc-3’ | 621 bp  55˚C |

**Supplementary Table S4.** **Primers for sequencing of *Lmnb1* exons**. All primers are flanking coding regions of the respective exons. Sizes of product-fragments are given in base pairs (bp), including primer sequence. T° indicates annealing temperature used for PCR amplification.
